# The effects of methylphenidate and atomoxetine on *Drosophila* brain at single-cell resolution and potential drug repurposing for ADHD treatment

**DOI:** 10.1038/s41380-023-02314-6

**Published:** 2023-11-13

**Authors:** Susu Qu, Xiangyu Zhou, Zhicheng Wang, Yi Wei, Han Zhou, Xinshuang Zhang, Qingjie Zhu, Yanmin Wang, Quanjun Yang, Likun Jiang, Yuan Ma, Yuan Gao, Lei Kong, Li Zhang

**Affiliations:** 1 Academy for Advanced Interdisciplinary Studies, Peking University, Beijing, China; 2 Chinese Institute for Brain Research, Beijing, China; 3 Department of Pharmacy, Shanghai Jiao Tong University Affiliated Sixth People’s Hospital, Shanghai, China; 4 Department of Computer Science, Xiamen University, Xiamen, China; 5 Center for Bioinformatics, State Key Laboratory of Protein and Plant Gene Research, School of Life Sciences, Peking University, Beijing, China

**Keywords:** Psychology, Cell biology, Molecular biology

## Abstract

The stimulant methylphenidate (MPH) and the non-stimulant atomoxetine (ATX) are frequently used for the treatment of attention-deficit/hyperactivity disorder (ADHD); however, the function of these drugs in different types of brain cells and their effects on related genes remain largely unknown. To address these questions, we built a pipeline for the simultaneous examination of the activity behavior and transcriptional responses of *Drosophila melanogaster* at single-cell resolution following drug treatment. We selected the *Drosophila* with significantly increased locomotor activities (hyperactivity-like behavior) following the administration of each drug in comparison with the control (same food as the drug-treated groups with 5% sucrose, yeast, and blue food dye solution) using EasyFlyTracker. Subsequently, single cell RNA sequencing (scRNASEQ) was used to capture the transcriptome of 82,917 cells, unsupervised clustering analysis of which yielded 28 primary cell clusters representing the major cell types in adult *Drosophila* brain. Indeed, both neuronal and glial cells responded to MPH and ATX. Further analysis of differentially expressed genes (DEGs) revealed distinct transcriptional changes associated with these two drugs, such as two well-studied dopamine receptor genes (*Dop2R* and *DopEcR*) were responsive to MPH but not to ATX at their optimal doses, in addition to genes involved in dopamine metabolism pathways such as *Syt1*, *Sytalpha*, *Syt7*, and *Ih* in different cell types. More importantly, MPH also suppressed the expression of genes encoding other neurotransmitter receptors and synaptic signaling molecules in many cell types, especially those for Glu and GABA, while the responsive effects of ATX were much weaker. In addition to monoaminergic neuronal transmitters, other neurotransmitters have also shown a similar pattern with respect to a stronger effect associated with MPH than with ATX. Moreover, we identified four distinct glial cell subtypes responsive to the two drugs and detected a greater number of differentially expressed genes associated with ensheathing and astrocyte-like glia. Furthermore, our study provides a rich resource of candidate target genes, supported by drug set enrichment analysis (*P* = 2.10E-4; hypergeometric test), for the further exploration of drug repurposing. The whole list of candidates can be found at ADHDrug (http://adhdrug.cibr.ac.cn/). In conclusion, we propose a fast and cost-efficient pipeline to explore the underlying molecular mechanisms of ADHD drug treatment in *Drosophila* brain at single-cell resolution, which may further facilitate drug repurposing applications.

## Introduction

Attention-deficit/hyperactivity disorder (ADHD) is a neurodevelopmental condition characterized by inattention, hyperactivity, and impulsivity, and its prevalence is approximately 7.2% worldwide [1] and 6.4% in China [2]. The pathogenesis of ADHD remains unclear and its etiology is complicated. Pharmacological interventions are effective in some patients with ADHD, including the stimulants MPH and amphetamine, and the non-stimulants ATX, extended-release clonidine, and guanfacine [3]. Most drug treatments for ADHD aim to regulate inter-synaptic neurotransmitter levels, and MPH and ATX are frequently used to treat ADHD since they help with the primary symptoms and cognitive dysfunction [4]. A reasonable mechanism of action for MPH and ATX would be the regulation of inter-synaptic neurotransmitter levels, since MPH inhibits the reuptake of norepinephrine (NE) and dopamine (DA) in presynaptic neurons by inhibiting norepinephrine transporters (NETs) and dopamine transporters (DATs) [5]; and ATX selectively inhibits presynaptic NET, having secondary effects on the dopaminergic system [6]. However, the “neurotransmitter regulation hypothesis” does not offer a satisfactory explanation for the current experimental or clinical findings. For instance, the extracellular concentration of DA in neuronal cell lines devoid of *DATs* is significantly decreased following treatment with MPH [7], indicating the inhibition of NETs or the existence of other possible targets. MPH also has a weak effect on the regulation of serotonin (5-HT) and glutamate (Glu), and even other general cellular processes [8, 9]; therefore, the underlying mechanisms of these ADHD medications require further investigation. It is noteworthy that different doses of ADHD drugs such as MPH have been shown to work differently, not only treating disease but also significantly increasing locomotor activity (hyperactivity-like behavior typical of ADHD) in the control group [10–12]. These drugs, originally approved to treat ADHD [13], produce the same paradoxical effects in humans as well as in rodent and fly models [14–17].

The manner by which ADHD drugs, such as MPH and ATX, regulate different cell types and related genes remains unknown. Due to the difficulties in acquiring human brain samples, it is challenging to tackle this question in humans; however, since approximately 75% of human disease-causing genes are evolutionarily conserved between humans and *Drosophila melanogaster* [18], this organism is widely used as a fast and cost-efficient model to study human disease [19]. Moreover, the central nervous systems of mammals and *Drosophila* are conserved in their evolutionary origin [20, 21]; for example, synapses between neurons share a common protein structure and neurotransmitter substances, including acetylcholine (ACh), gamma-aminobutyric acid (GABA), Glu, DA, and 5-HT. Additionally, octopamine (OA) is the invertebrate homolog of mammalian NE and plays important roles in the modulation of behavior and synaptic functions [22, 23]. Furthermore, emerging evidence indicates that distinct subtypes of glial cells play a crucial role in the control of neuronal development, apoptosis, metabolism, sleep, and other physiological activities in *Drosophila* [24]. The presence of diverse but well-conserved cell types in *Drosophila* provide an excellent tool to investigate the differential responses of different cells to drugs.

Most importantly, studies have shown that knockdown or knockout of target homologous genes (e.g., *GARNL3*, *SLC6A3*, *LPHN3*, *NF1*, *MEF2C*, and *TRAPPC9*) in *Drosophila* results in ADHD-like behaviors (attention deficit or hyperactivity) that can be rescued by MPH treatment [25–28]. We previously developed the user-friendly software EasyFlyTracker [29], which enables large-scale tracking and analysis of the sleep/locomotor activity of drug-treated *Drosophila*. Additionally, the development and widespread use of single cell RNA sequencing (scRNASEQ) technology has increased our understanding of the cellular composition of many tissues in *Drosophila* [30], including the aging brain [31], larval brain [32], adult midbrain [33], and adult midgut [34], as well as the effects of single-cell level changes in *Drosophila* brain under acute cocaine [35] and alcohol [36] exposure. The effects of MPH and ATX treatment on *Drosophila* behavior and cell type-specific transcriptional changes throughout the brain have not yet been reported. Choosing to treat wild-type *Drosophila* rather than those with aforementioned ADHD-like behavior [25–28] or existing rodent models [17], which usually knockout or knockdown certain ADHD-related genes, may identify comprehensive drug responsive genes as a baseline and avoid biases introduced by disease models that differ from ADHD patients. Thus, a complete cell atlas containing cell type-specific responsive gene sets would likely provide a theoretical basis for the further exploration of ADHD treatment. Since our animal model may differ from human ADHD patients, we employed the drug set enrichment analysis to confirm enrichment of ADHD drugs in our results.

Drug-responsive gene sets potentially contain ADHD drug targets, especially those already known to be druggable [37]. Indeed, the number of potentially druggable genes has increased [38] beyond those originally estimated according to fundamental pharmacological principles [37]. Druggable genes have been defined by Finan et al. as a set of 4479 genes, and divided into three tiers based on druggability level. Tier 1 contains targets of approved small molecules and biotherapeutic drugs as well as those in clinical trials [38]. In combination with these druggable genes, a framework for assessing the druggability of ADHD [39] has been proposed with the aim of elucidating new avenues for the development and reuse of ADHD drugs. De novo discovery and development of entirely new drugs targeting the unique biology of a disease is a long and expensive process with a low success rate; therefore, it is economically efficient to use existing drugs for new indications. ATX, which was approved in November 2002 [40], is a famous example of a successful repurposed drug. Recently, several studies have focused on exploring further targets or repurposing drugs [41] for cognitive (nootropics) diseases [42], Parkinson’s disease [43], and schizophrenia [44]. Following the same logic, we expect to provide a list of potential repurposed drugs for further exploration based on the identified gene sets.

Here, we performed behavioral studies in adult male *Drosophila melanogaster* exposed to MPH, ATX, and control treatments. Subsequently, we carried out scRNASEQ on whole brain with hyperactivity-like phenotypes following drug administration in comparison with the control (same food as the drug-treated groups with 5% sucrose, yeast, and blue food dye solution), yielding various cell types that responded to these drugs. In total, we identified 28 distinct cell clusters including neurons and glia and provide single-cell resolution gene expression data following MPH and ATX treatment. Based on the primary cell atlas, we analyzed shared genes and pathways between MPH and ATX treatment, and neurotransmitters with a related hypothesis are explored and discussed. Glial cells were also found to be affected. In addition, the expression patterns of cytochrome P450 (CYPs) genes between clusters are summarized to reveal drug effects, since CYP genes are important in drug metabolism and clinical response. Moreover, we examined cell–cell communication between clusters to explore drug effects on the signaling between neurons and glial cells. Furthermore, we analyzed the relationship among ADHD candidate genes, FDA-approved ADHD drug target genes, and the homologs of these drug-responsive genes in *Drosophila*. By identifying potential new drug sets using the druggable genome, we provide additional possible targets and opportunities for potential drug repurposing. In addition, we conducted drug set enrichment analysis (hypergeometric test) to confirm that the repurposing results found by our approach are indeed relevant to ADHD. Finally, we built the ADHDrug website (http://adhdrug.cibr.ac.cn/) that enables users to view scRNASEQ data, query specific gene expression in various cell types, and search the list of potential repurposing drugs. In the present study, we propose a new approach for the rapid and cost-effective investigation of the response to MPH and ATX in *Drosophila* models at single cell resolution. This approach, although not suitable for immediate clinical translation, may serve as a novel pipeline for potential drug repurposing for ADHD.

## Results

### Both MPH and ATX increase the locomotor activity of wild-type *Drosophila*

**Fig. 1:**
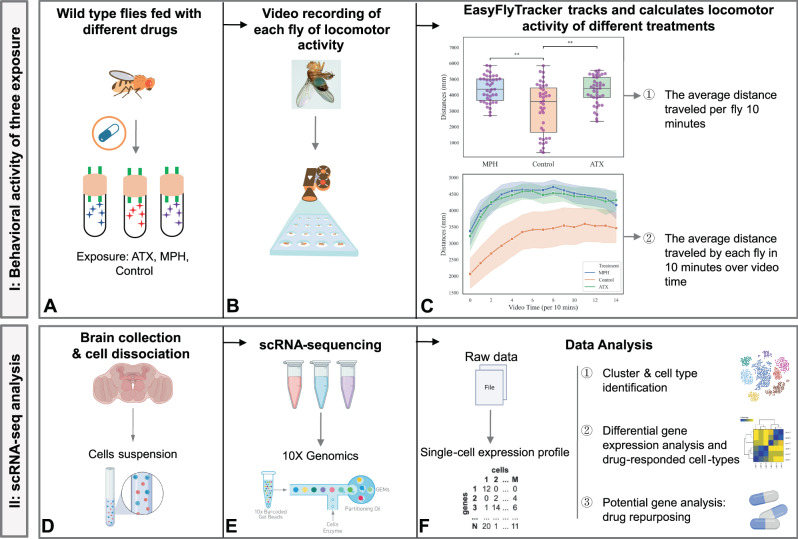
Workflow of the psychotropic drug-exposed scRNASEQ study in adult *Drosophila* brain. The workflow of our drug-exposed scRNASEQ study contains two parts. The first part (shown in subfigure **A**–**C**) is to conduct a behavioral activity assay in wild-type flies using methylphenidate (MPH) and atomoxetine (ATX) to generate hyperactivity-like behavior, which is video recorded and subsequently used to analyze locomotor activity. The second part (shown in subfigure **D**–**F**) is to conduct single-cell RNA sequencing and data analysis, which aims to reveal the effects of the aforementioned drugs at single-cell resolution. **A** Wild-type flies were fed with different drugs (ATX, MPH, or control) using a modified capillary feeder assay (CAFE). **B** The locomotor activity of each fly was recorded by camera under different conditions, and each batch was recorded for 2.5 h. Two experiments were performed separately on July 12th (replicate 1) and August 10th 2021 (replicate 2). **C** Analysis of the video recordings. EasyFlyTracker tracked and calculated the locomotor activity of different treatments. There are two main metrics used to calculate the locomotor activity: ① the average distance traveled per fly in a 10-min time period (2 replications); and ② the average distance traveled by each fly in a 10-min time period at each time point during the entire video. Both were significantly (** 0.001$<$ *P* $\le$0.01) increased in the MPH-treated or ATX-treated groups as compared with the control group throughout the 2.5-h video. **D** A total of 20 male fly brains were collected and dissociated to generate single-cell suspensions for each sample. Each replicate contained three samples exposed to MPH, ATX, or control treatment. **E** After cell dissociation, counts larger than 500 live cells/μL were used to prepare 10X Genomic sequencing libraries. **F** Analysis of scRNASEQ data for all samples contains three different parts: ① clustering and cell type identification; ② differential gene expression analysis and drug-exposed cell type identification; and potential gene analysis for drug repurposing.

To investigate the cell type-specific molecular mechanisms of ADHD drugs in the brain at single-cell resolution, we conducted behavioral experiments and scRNASEQ in wild-type (WT) adult male *Drosophila melanogaster* following exposure to MPH, ATX, and control treatment. Here, we chose WT flies to identify comprehensive drug responsive genes rather than using the existing models of ADHD-like behavior in fruit flies [25–28] or rodents [17], which usually knockout or knockdown certain ADHD-related genes. Only WT male flies that displayed hyperactivity-like behavior (significantly higher locomotor activity) after drug treatment in comparison with the control were selected for sequencing. Subsequently, we dissected and dissociated whole brains under three different conditions, captured the phenotypes after drug administration, and subjected single cells to 10X Genomics scRNASEQ. An overview of the workflow of the drug-exposed scRNASEQ study in adult *Drosophila* brain is shown in Fig. 1. MPH and ATX are commonly used drugs to treat ADHD symptoms in humans [3]. In the modified capillary feeder (CAFE) assay [45], WT male flies were exposed to MPH, ATX, and control treatment for 24 h (Fig. 1A). After selecting a dose according to the literature [10], the drugs were tested at four or five different doses (for ATX: 0.25 mg mL^-1^, 0.5 mg mL^-1^, 1 mg mL^-1^, and 2 mg mL^-1^; for MPH: 0.25 mg mL^-1^, 0.5 mg mL^-1^, 1 mg mL^-1^, 1.5 mg mL^-1^, and 2 mg mL^-1^) to find the inflection point of the dose-response curves. The results show that 0.25 mg mL^-1^ ATX (Supplementary Fig. 1A) and 1.5 mg mL^-1^ MPH (Supplementary Fig. 1B) had the strongest effect; therefore, these concentrations were chosen for subsequent experiments. A single adult fruit fly was placed in each arena after drug exposure (a total of 24 flies per treatment, and 72 flies overall) and its behavior was recorded (Fig. 1B). Two distinct replications were carried out on July 12th (replicate 1) and August 10th (replicate 2) 2021. The locomotor activities of fruit flies were simultaneously tracked using EasyFlyTracker and the short-term distances were quantitated. Only 60 out of the 72 flies (20 flies per treatment) were used for subsequent behavioral calculations and experiments. We found that WT male flies produced hyperactivity-like behavior (higher locomotor activity) following exposure to MPH or ATX in comparison with the controls, as shown in Fig. 1C. Additionally, we observed a significant increase (Fig. 1C ①) in the average distance traveled per fly in a 10-min time period in MPH-exposed (Kruskal–Wallis test with Bonferroni correction: *P* = 1.463E-03) or ATX-exposed (Kruskal–Wallis test with Bonferroni correction: *P* = 3.766E-03) flies, which is consistent with previously published results [10, 26]. Moreover, the line plot of the average distance traveled by each fly at each time point during the 2.5-h recording period also shows markedly higher-level activities in drug-exposed groups as compared with control flies (Fig. 1C ②). Furthermore, EasyFlyTracker created angle-change plots (Supplementary Fig. 1C) and heatmaps (Supplementary Fig. 1D) of the different treatments to display more details of the behavioral activities of the fruit flies. We found a positive correlation (Pearson *r* of distances and angles in all groups: 0.6979, *P* = 6.71E-263) between the pattern of angle-change activities and locomotor activities in both the drug-exposed and control groups, as shown by the scatter plot in Supplementary Fig. 1C.

### scRNASEQ identified 28 distinct primary clusters in adult male *Drosophila* brain

**Fig. 2:**
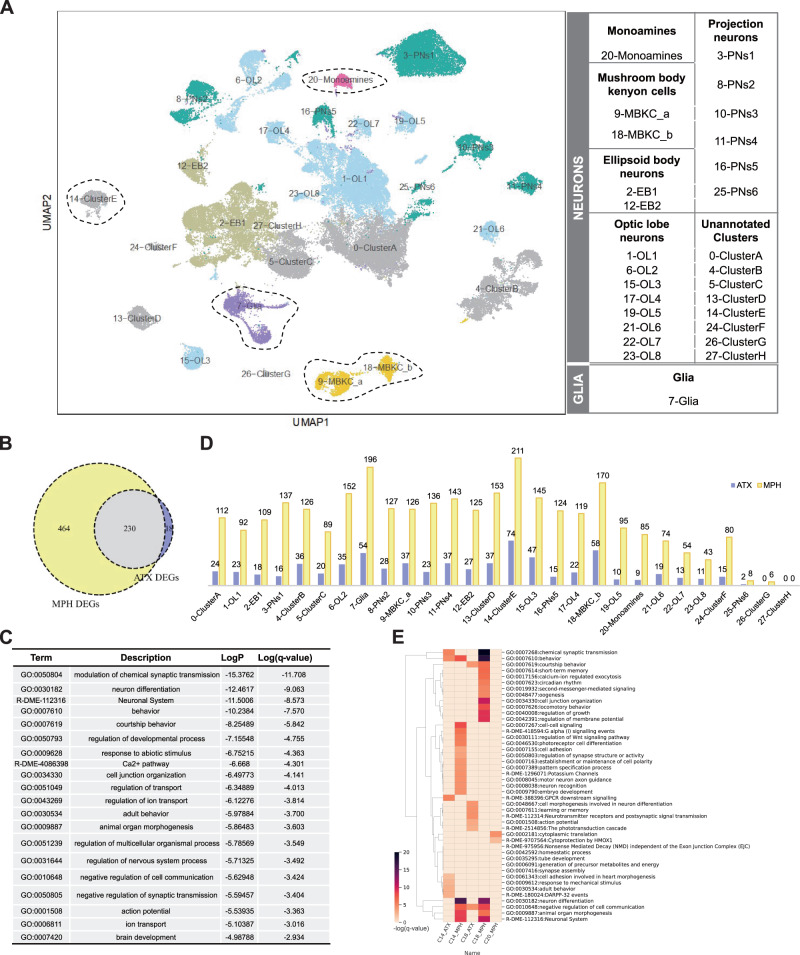
Cell atlas of adult *Drosophila* brain and the biological pathways responsive to MPH and ATX. **A** UMAP clustering and visualization of scRNASEQ data. Cells were clustered based on their expression pattern using the unsupervised shared nearest neighbor (SNN) clustering algorithm. Individual dots represent a single cell, and the color of the dot represents the cluster to which the cell belongs. Identification of cell types from clusters (shown in the right panel) was performed by canonical marker and top gene annotation from the literature. Clusters were classified based on function. **B** Venn diagram showing the numbers of total, unique, and shared DEGs (logfc.threshold = 0.25 & Bonferroni-adjusted *P* $\le$ 0.05) following MPH and ATX treatment. **C** Shared pathways of the common DEGs between MPH and ATX treatment. **D** Statistics for the number of DEGs in each cluster following MPH and ATX treatment. **E** Pathway analysis of selected neuronal clusters (C14, C18, and C20) by Metascape. Color bar represents the -log(*q*-value) of the pathway calculated using Metascape.

We dissected the brains from WT male fruit flies in the MPH-treated, ATX-treated, and control groups following the observation of significant hyperactivity-like behavior in comparison with the controls (Fig. 1D). A total of 60 male flies were dissected for one behavioral test (20 brains per treatment). Subsequently, three independent samples from each batch were processed for single-cell isolation and the mRNAs were barcoded and sequenced (Fig. 1E). We analyzed the sequencing data at different levels, as shown in Fig. 1F. Since the number of recovered cells was greater than expected, DoubletFinder [46] was used to predict and remove doublets. Details of the number of cells and other statistics are summarized in Supplementary Table 1. A total of 82,917 cells were retained for subsequent analysis. We primarily identified 28 distinct clusters at low resolution (0.1; 15 PCA), annotated the clusters based on previous understanding of canonical markers and the top 10 marker genes in each cluster, and were able to clearly distinguish between neurons and glial cells. These marker genes are summarized in Supplementary Table 2, and the preliminary visualization of the cell type annotation is shown in Fig. 2A. At first, neurons and glial cells were roughly regarded as two main types according to the classical marker genes *elav* and *repo*, and then detailed cell types were annotated according to Supplementary Table 2, including monoaminergic neurons (Monoamines), mushroom body Kenyon cells (MBKCs), ellipsoid body cells (EB), optic lobe cells (OL), projection neurons (PNs), unannotated clusters that cannot be identified according to the primary classification (Clusters A–H), and glial cells (Glia). It is well known that mushroom bodies, which contain three subclasses of neurons, $\alpha \beta ,{\alpha }^{{\prime} }{\beta }^{{\prime} },{{{{{\rm{and}}}}}}\,\gamma$, are essential for olfactory learning and memory. Using the well-known markers *ey* and *Dop1R2*, in addition to the other top 10 marker genes listed in Supplementary Table 2, we were able to directly distinguish between two different MBKC types. As shown in Fig. 2A, only a small fraction of cells expressed the known marker gene Vesicular Monoamine Transporter (*Vmat*), which were independently marked as monoaminergic neuronal cells. No obvious sub-clusters of Monoamines (C20) were found according to known marker genes corresponding to each of these neurons releasing 5-HT, tyramine (TA), octopamine (OA), and DA in *Drosophila* (details can be found in the Methods); thus, we regarded Monoamines (C20) as our research target representing dopaminergic neurons. Certain clusters related to the hypothesis or mechanism of drugs were fully considered and analyzed. The number of differentially expressed (drug-responsive) genes between the treatment and control groups was calculated for each primary cell type.

### The general effects of MPH and ATX in neurons and glial cells

After careful annotation of the cell types, we used Seurat’s default parameters (logfc.threshold = 0.25 and Bonferroni-adjusted *P* $\le$ 0.05) to identify DEGs. We identified many drug-responsive DEGs across all clusters: 694 for MPH and 248 for ATX, with 230 genes shared between the two groups as shown by the Venn diagram (Fig. 2B). Previous studies have shown that both drugs greatly enhance the cognitive function and symptoms of ADHD, which raises the possibility that they share overlapping mechanisms of action [4, 47–49]. Thus, the biological pathways of the 230 genes shared between MPH and ATX were analyzed, and the top 20 pathways are displayed in Fig. 2C. These top common pathways include those that modify chemical synaptic transmission, the Ca^2+^ pathway, the negative regulation of synaptic transmission, ion transport, the neuronal system, adult behavior, and the regulation of cell–cell communication. The full set of results can be found in Supplementary Table 3. These pathways are mainly associated with the regulation of neurotransmitters, which is in agreement with the general hypothesis of ADHD as a neurotransmitter disorder.

More specifically, a wider range of cell type responses was identified for the stimulant MPH in comparison with the non-stimulant ATX after analysis of DEGs in individual cell types (Fig. 2D). Exposure to MPH and ATX induced widespread changes in gene expression throughout the brain, with a stronger effect associated with MPH. The widespread “neurotransmitter imbalance” hypothesis was evaluated by focusing on the Monoamine (C20) cluster that explicitly expresses *Vmat*. Additionally, the top three clusters containing the greatest number of DEGs were Cluster E (C14), Glia (C7), and MBKC_b (C18), respectively. We performed pathway analysis of the neuronal cell types C20, C14, and C18 to identify cell type-specific signals, as shown in Fig. 2E (C7 was introduced later in “Subdivision of glial cells and their essential role in MPH and ATX effects”). The full plot of the biological pathways is shown in Supplementary Fig. 2. We found that the cell type-specific enrichment pathways are mostly implicated in MPH effects. For instance, C20 shows a limited number of enrichment pathways concentrated in MPH, with none in ATX. Moreover, these cell types perform different functions in the MPH-treated and ATX-treated groups, sharing only a limited number of pathways in *Drosophila* brain. For example, these cells only share two pathways (negative regulation of cell communication and courtship behavior) in C18, and the rest are related to MPH. C18 is the second-highest cluster, which is a subtype of MBKC and essential for olfactory learning and memory. MBKC forms numerous synapses with DA neurons, and recent results have highlighted the importance of DA-driven plasticity and activity in feedback and feedforward connections between various elements of the mushroom body neural networks [50]. Although we do not yet have the exact cell type mapping between *Drosophila* and humans, the different responses to MPH and ATX in *Drosophila* brain cell types further support the diversity of drug responses and the importance of precise treatment.

### Neurotransmitter-related gene expression pattern in adult *Drosophila* brain at single-cell resolution

**Fig. 3:**
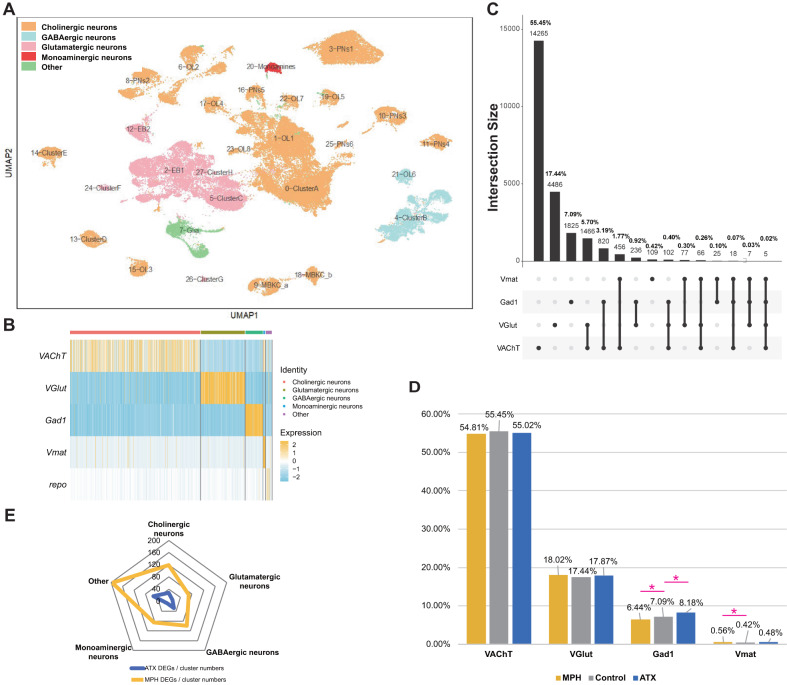
Proportion and distribution analysis of neurotransmitters in adult *Drosophila* brain. Control samples from two replicates were combined with drug-treated samples to analyze the proportion and distribution of neurotransmitters. **A** DimPlot of Seurat based on UMAP displaying the distribution of cell types from the primary classification. Cells were classified based on the expression of the marker genes of neurotransmitters and are shown in different colors. **B** Simplified heatmap showing one glial and four neurotransmitter populations. Displayed genes are the main markers analyzed to identify neuronal and glial cell identities. The horizontal axis represents individual cells; each line corresponds to one cell. Gene expression levels are coded by color intensity. **C** UpSet plot [152] illustrating the co-expression of neurotransmitters in neuronal cells. Light and bold numbers represent the number and percentage of cells, respectively. The effects of MPH and ATX treatment were considered based on the integrated samples. **D** Proportion of different neurotransmitters in neuronal cells following treatment with MPH (yellow), control (gray), and ATX (blue). The Fisher Exact probability test was used to detect significant differences between the drug groups and the control group by R v4.1.0 (prop.test()), the parameters of which were as follows: alternative = “two.sided”, conf.level = 0.95, correct = TRUE. Significant changes are marked with an asterisk (*). **E** Radar plot showing the average number of DEGs (DEGs/cluster numbers) in the different groups following MPH (yellow) and ATX (blue) treatment.

Prior knowledge demonstrates that the different neurotransmitter hypotheses are essential to the pharmacological treatment of psychiatric diseases [51–54]. Here, neurotransmitters can be analyzed at both the cell type and DEG level at single-cell resolution. We firstly assessed the proportion and distribution of cells expressing genes responsible for the release or synthesis of different neurotransmitters in *Drosophila* brain, which provides valuable information to fully understand the mechanisms induced by the two drugs. We classified cells as glutamatergic, cholinergic, GABAergic, and monoaminergic neurons based on the expression of key genes, *vesicular glutamate transporter (VGlut)* of Glu, *vesicular acetylcholine transporter (VAChT)* of ACh, *Glutamic acid decarboxylase 1 (Gad1)* of GABA, *or vesicular monoamine transporter (Vmat)* of monoamines, and *repo* of glial cells were classified as other type. These cells are plotted in different colors in Fig. 3A, and the aforementioned names of the primary cell types are also labeled to elaborate. Fortunately, we did not observe any detectable expression of neurotransmitter markers in glia, as shown in the heatmap in Fig. 3B. Cholinergic neurons were found to be the most abundant in the control samples, being expressed in 55.45% of all cells; however, glutamatergic, GABAergic, and monoaminergic neurons were expressed in only 17.44%, 7.09%, and 0.42% of cells, respectively (Fig. 3C). Since recent research in *Drosophila* revealed a list of co-expressed neuroactive substances [32, 33], we also looked at the possibility of co-existing neurotransmitters in the adult male brain. As shown in Fig. 3C, cells expressing these neurotransmitter-specific marker genes were mainly exclusive, despite the presence of 5.70% *VAChT* and *VGlut* indicators and 3.19% *VAChT* and *Gad1* markers. Some cells simultaneously release both excitatory and inhibitory neurotransmitters. This phenomenon shows that co-expression of excitatory and inhibitory neurotransmitters also occurs in the adult male *Drosophila* brain, which is consistent with scRNASEQ data for larval brain [32] and midbrain [33]. The percentage of cells expressing the markers for Glu, GABA, or all three or four neurotransmitters was very low (<1%) (Fig. 3C), despite the possibility that these markers represent multiple cells. Analyzing the distribution and proportion of these neurotransmitters can aid our understanding of the cellular and molecular processes related to ADHD drugs.

Even though the pathophysiology of ADHD remains largely unknown, the neurotransmitter imbalance hypothesis has been continuously described; thus, cell type proportions of the “neurotransmitter levels” induced by MPH and ATX treatment were quantitated according to the expression levels of key genes. As shown in Fig. 3D, the proportions of cell types induced by drug treatment changed only slightly, indicating that the significant effects (marked with *) on GABAergic and monoaminergic neurons only affected the gene expression level of a small number of cells. Specifically, we found that monoaminergic neurons only changed significantly following MPH treatment (Fisher Exact probability test: *P* = 2.89E-02), GABAergic neurons changed significantly following both MPH (Fisher Exact probability test: *P* = 2.13E-03) and ATX (Fisher Exact probability test: *P* = 5.30E-06) treatment, while changes in other neurons (cholinergic and glutamatergic) were non-significant. These may be drug-induced changes in the expression of certain genes or a result of the “neurotransmitter switch”. Neurotransmitter switching, the gain of one transmitter and the loss of another in the same neuron, can be driven by natural stimuli, drugs, and other programs used to manage neurological and psychiatric disorders that also affect neurotransmitter states and thus alter behavior, which has been observed in previous studies [55]. Our results provide the possibility for further research to reveal the manner by which psychotropic drugs alter key gene expression and neurotransmitter switching in certain cells in the brain. Moreover, trends (although non-significant) in excitatory glutamatergic neurons increased following both MPH and ATX treatment in comparison with the control; however, excitatory cholinergic neurons decreased and inhibitory GABAergic neurons changed in different directions following MPH and ATX treatment. The imbalance between excitation and inhibition is associated with ADHD-like symptoms and drug-induced mechanisms. A previous study has shown that these imbalances may contribute to the development of ADHD-like phenotypes in a mouse model [56]. Moreover, the distinct direction of change following MPH and ATX treatment indicates that the underlying molecular mechanisms are different. Indeed, gene set association analysis in humans has revealed that Glu, and possibly also GABA, are associated with ADHD and ASD, although the direction of the effects remains undetermined [57]. Next, we evaluated the difference in expression levels of neurotransmitter marker genes (*VAChT*, *VGlut*, *Gad1*, *Vmat*, and *repo*) between the drug treatment and control groups and found that *Gad1* was significantly expressed following treatment with both drugs (Wilcox test of MPH: *p_val_adj* = 5.47E-41, Wilcox test of ATX: *p_val_adj* = 3.28E-51), which is the common candidate gene for both drugs. Additionally, we visualized the patterns following treatment with MPH and ATX by counting the normalized DEGs in the five groups of cells using a radar plot, as shown in Fig. 3E. We discovered similar neurotransmitter expression levels but different expression intensity of the normalized DEGs between the MPH-treated and ATX-treated groups for most cell classes, with the exception of monoaminergic neurons in which ATX treatment induced a lower number of DEGs in comparison with MPH. However, cell type groups such as cholinergic, glutamatergic, and GABAergic neurons, and even glial cells, showed similar expression preferences. These results support the notion that MPH is a much broader neurotransmitter inhibitor. Taken together, monoaminergic neurons react to both MPH and ATX treatment in distinct ways; for example, in the same direction but with different intensity.

In summary, both MPH and ATX treatment affects different neurotransmitter neurons, producing a slight change in cell type proportions. Since the chance of changes in cell type proportions is small, it is more likely that drug treatments have a significant effect on gene expression in a small proportion of cells, resulting in gene expression changes or neurotransmitter switching. Moreover, common effects of drug treatment were observed. At the cellular level, the proportion of different neurotransmitters in cholinergic and glutamatergic cells following treatment with MPH and ATX were not significantly different, while both displayed significant changes in GABAergic cells. At the gene level, responsive genes such as *Gad1* deserve further investigation.

### Dopamine metabolism and signaling respond to MPH and ATX

As mentioned previously, neurotransmitters are thought to be critical in the field of ADHD research, especially monoaminergic neurons. Catecholamines (DA, NE), 5-HT, and GABA display dysfunction or deficit in ADHD [58–61]. Most drug treatments for ADHD, such as MPH [5] and ATX [6], aim to regulate inter-synaptic neurotransmitter levels. The enhanced efflux of DA and NE associated with MPH or ATX exposure leads to increased availability for binding to their respective transporters (such as the DAT and NET) and receptors, as evidenced by existing studies [12, 62–65]. Here, we aim to summarize the drug responses of DA and NE, by using OA in *Drosophila* to replace NE, which is the invertebrate homolog of mammalian NE and plays important roles in modulating behavior and synaptic functions [22, 23]. DA metabolism and signaling is discussed in this section, and the results for OA are shown in the subsequent section.

**Fig. 4:**
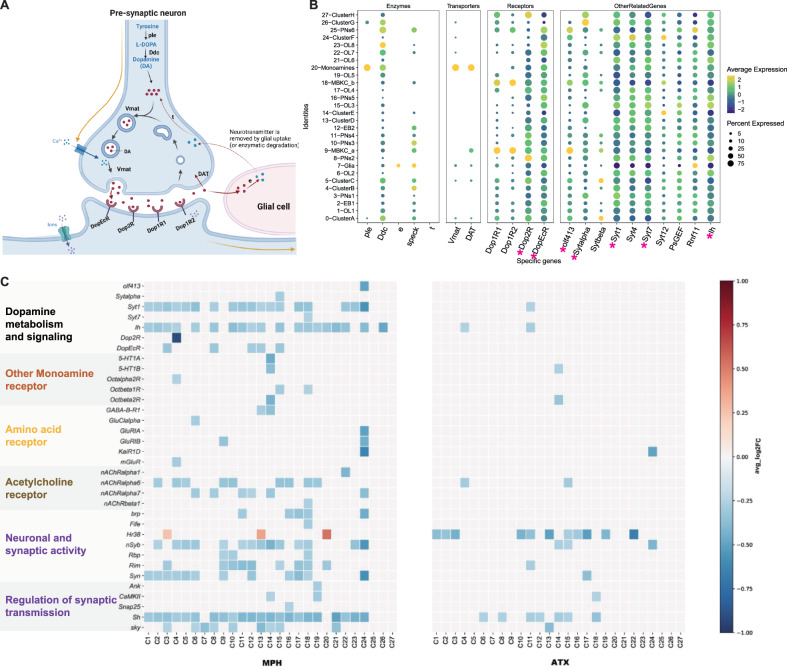
Identification of genes and cell types involved in the psychotropic drug hypothesis. Dopamine metabolism and signaling was analyzed first, and receptor genes for other neurotransmitters related to the hypothesis are shown later. **A** Major proteins involved in the metabolism and signaling of dopamine (DA) are shown in the schematic of a dopaminergic synapse. The “Chemical Synapse: Steps of Synaptic Transmission” template was used to create the figure, which was then modified using drawings from Yamamoto (2014) and Croset (2018) and concatenated using BioRender.com (2022). **B** DotPlot showing the average expression and percentage of expression of specific genes involved in DA metabolism and signaling in all clusters. **C** Heatmap showing the fold change in specific DEGs following MPH (left panel) and ATX (right panel) treatment in some specific cell types. Functional groups of genes are indicated with the same color. DEGs ( | log2FC | $\ge$ 0.25, Bonferroni-adjusted *P* $\le$ 0.05) are represented by the rows and cell clusters are listed at the bottom (columns). Color bar shows the direction of gene expression regulation following exposure to drugs, and the values inside the box indicate the log2FC.

DA signaling is regulated by enzyme degradation and transporter reuptake, and the recycled metabolites can be reused to synthesize DA (Fig. 4A). These steps can occur in different cell types, such as DA-releasing cells, postsynaptic neurons, and glial cells [66]; therefore, we used our scRNASEQ data to determine which cell types expressed components of the DA recycling and metabolic pathways. The first step of DA synthesis, conversion of tyrosine into the DA precursor L-DOPA catalyzed by the *ple*-encoded Tyrosine hydroxylase, appears to primarily occur in Monoamines (C20) as compared with other cell clusters (Fig. 4B). In comparison, *Ddc*, which converts L-DOPA to DA, is present in several other neuronal populations, including OLs, PNs, and other non-specific clusters (Fig. 4B). It is unclear whether *Ddc* present in these neurons is involved in the metabolism of DA and other aromatic L-amino acids; however, these two genes were not significantly differentially expressed among the MPH, ATX, and control treatments. Three enzymes play a role in DA degradation and recycling (Fig. 4A). Firstly, the *ebony* (*e*) gene product converts DA into N-beta-alanyl-dopamine (NBAD) [67, 68] and was almost exclusively expressed in glial cells, but only occupied 25% in our data (Fig. 4B). Secondly, Dopamine-N-acetyltransferase, encoded by *speck*, converts DA into N-acetyl-dopamine (NADA). *speck* was expressed in glial cells, PNs, and non-specific cell types (such as C0, C4, and C5) (Fig. 4B). Although these results highlight the important role of glia in DA reuptake, metabolism, and recycling, other cells appear to convert DA into NADA rather than into NBAD (Fig. 4A, B). The fate and consequence of these two metabolites in each cell type remain largely unknown. Thirdly, *tan* (*t*), a gene coding a hydrolase that can convert NBAD back to DA, was nearly not found in any cell population from the brain itself (Fig. 4B), suggesting that this recycling pathway is not utilized there. Nevertheless, these three genes were also not significantly differentially expressed among MPH, ATX, and control treatments.

The vesicular monoamine transporters (encoded by *Vmat*) transport DA, 5-HT, OA, and TA into synaptic vesicles [69]. As already mentioned, *Vmat* was mainly detected in Monoamines (C20). The *DAT*-encoded DA transporter mediates DA reuptake by dopaminergic neurons. Unlike *Vmat*, *DAT* was not only specifically expressed in dopaminergic cells, but also found in MBKCs (C9-MBKC_a” cluster in Fig. 4B), suggesting that other neurons may tightly regulate the duration and magnitude of DA signals that they receive. However, these two transporters were also not significantly differentially expressed among MPH, ATX, and control treatments. These results are not consistent with the previously discussed hypothesis that MPH acts primarily by inhibiting *DAT* [70]. Here, we propose several reasons for this discrepancy. Firstly, the high dose may be one reason for the non-significant differential expression of *DAT* following MPH treatment, since previous research has shown that different doses of MPH can lead to different results [11]. For example, one study found that a high dose of MPH appears to suppress intracranial self-stimulation through mechanisms other than DAT inhibition [71]. Although we used the inflection point of locomotor activities (as shown in Supplementary Fig. 1A, B), which followed previous experiments with MPH [10, 26], no such experiments have been performed in *Drosophila* with ATX; thus, it is difficult to describe the dose effects of MPH and ATX in our experiments. Secondly, structural biology research has demonstrated that *Drosophila DAT* possesses differences in subsite B of the central binding site as compared with human *DAT*, which leads to much weaker inhibition of the stimulant amphetamine in humans [72]. Thirdly, it has been shown that the extracellular concentration of DA is significantly decreased in neuronal cell lines devoid of *DAT* following treatment with MPH [7]. Therefore, we suggest that these factors prevent us from viewing the pattern of *DAT* inhibition.

In addition to *DAT*, our data also explore the distribution and drug response of DA receptors, which are another important factor in the DA signaling pathway that influences DA levels in the synaptic cleft. As shown in Fig. 4B, DA receptors (*Dop1R1*, *Dop1R2*, *Dop2R*, and *DopEcR*) are not only distributed in MBKCs that form numerous synapses with DA neurons in the lobes of mushroom bodies, but also in Monoaminergic (C20) and projection neurons. Although we are unable to directly map the cell types between *Drosophila* and humans, various cell types in *Drosophila* brain expressed DA receptors and responded to the drugs, emphasizing the various cellular responses of DA. Our data demonstrate that *DopEcR* is expressed in almost all cell types, even glia, which is consistent with a previous study reporting this as an important receptor broadly expressed in *Drosophila* brain [73]. *DopEcR* is activated by DA as well as ecdysteroids (ecdysone and 20E) to increase cAMP levels and modulate multiple signaling cascades such as the phosphoinositide 3-kinase pathway [74, 75]. Other receptors, *Dop1R1* and *Dop1R2*, were mainly expressed in MBKCs, which has also been reported previously [76, 77]. We found that *Dop2R* and *DopEcR* were differentially expressed between the MPH-treated and control groups, but not the ATX-treated group. We distinguished *DopEcR* as the DEG in PNs (C3 and C8), EB2 (C12), Cluster D (C13), OL3 (C15), and MBKC_a (C9), and distinguished *Dop2R* in Cluster B (C4), as displayed in Fig. 3C. Since C4 lacks a direct location marker, we did not initially assign a specific cell type; however, we subsequently found that C4 had a high expression level of *Gad1* and thus annotated these cells as GABAergic neurons (Fig. 4A). These results revealed that the DA receptor *Dop2R* responded in GABAergic neurons. *Dop2R* encodes a G protein-coupled receptor that is activated by DA and regulates various phenotypes such as locomotor activity and olfactory associative learning. More importantly, the human ortholog of this gene, *DRD2* (dopamine receptor D2, DIOPT v8.0 score = 9), is implicated in several diseases including ADHD, conduct disorder, and movement disease. For example, single nucleotide polymorphisms (SNPs) in *DRD1* and *DRD2* are considered potential risk factors for ADHD [78], suggesting that the target of MPH is not only *DAT*; and more importantly, our results support an important role for DA receptors in MPH treatment. In addition to the above-mentioned common enzymes, transporters, and receptors, other genes are also involved in DA metabolism and signaling, as shown in Fig. 4B. Significant differential expression of these genes in at least one cell type is marked with an asterisk. These nine genes are all involved in the regulation of the DA secretory pathway of GO:0014059. As summarized in Fig. 4C, *Syt1*, *Sytalpha*, *Syt7*, and *Ih* were significantly differentially expressed in some specific clusters between the MPH-treated and control groups; *Sytalpha* was only significantly differentially expressed in OL3 (C15), *Syt7* only in MBKC_b (C18), and *Syt1* and *Ih* in various unannotated neuronal clusters (C0, C13, C14), PNs (C10, C11), EB2 (C12), OLs (C15, C17), and MBKC_b (C18); only *Syt1* (PNs (C11), C14 (ClusterE)) and *Ih* (ClusterB (C4), PNs (C11), C14 (ClusterE)) were significantly differentially expressed between the ATX-treated and control groups.

In summary, genes involved in DA metabolism and signaling pathways were detected in different cell types in adult *Drosophila* brain, such as MBKC, EB, OLs, PNs, and some unknown cell types, reflecting the diversity of drug effects. Our findings also support the previous view that DA receptors are crucial in ADHD: *Dop2R* and *DopEcR* were differently expressed between the MPH-treated and control groups, while there was no difference in the ATX-treated group. Additionally, genes involved in the regulation of the secretory pathway of DA, such as *Syt1*, *Sytalpha*, *Syt7*, and *Ih*, were mainly responsive to MPH treatment, suggesting the existence of more targets for the two drugs in *Drosophila* and the requirement for further exploration in humans. Here, the expression levels of DA-related signaling genes were clearly different between the ATX-treated and MPH-treated groups, highlighting a difference in the underlying mechanism that produces hyperactivity (higher locomotor activities) at the current dose, which may provide potential candidate targets for disease research. Moreover, these findings suggest that additional ADHD drug-responsive genes can be mined, and prospective candidates should be applied to other species such as mice, rats, and humans for further exploration.

### Genes for other neurotransmitter receptors and synaptic proteins also respond to MPH and ATX

It has been reported that MPH not only plays a role in regulating the DA pathway, but also NE, 5-HT, Glu, and even other more general cellular processes [8, 9]. Moreover, our previous evidence suggests that MPH may have multiple dimensional targets, such as receptors for different neurotransmitters. Thus, in addition to DA receptor genes, we also describe several drug-induced receptor changes for both MPH and ATX. We found that many cell types also significantly expressed the receptor genes for OA (similar to NE in humans), 5-HT, GABA, Glu, and acetylcholine (Fig. 4C and Fig. 6B), the patterns of which are summarized in Fig. 4C.

There was a lower expression level of all three OA receptors in the MPH-treated group, but only *Octbeta2R* was expressed at a lower level in the ATX-treated group. As previous research has shown, ATX increases the extracellular levels of NE, for which NET is its target [6]. ATX has a high affinity and selectivity for NET, but little-to-no affinity for other neurotransmitter transporters or receptors [6, 79]. Our results in *Drosophila* brain demonstrate the sparse inhibitory targets of ATX for OA and even other receptor genes, which is similar to previous results for NE. Promoted by the similar pattern of MPH and ATX at *Octbeta2R*, we found that it enables the activity of the OA receptor in *Drosophila* and that OA produces specific biochemical responses such as increased synthesis of cyclic AMP (cAMP) and phosphorylase activation [80]. *Octbeta2R* also participates in the adenylate cyclase-activating G protein-coupled receptor signaling pathway and positively regulates synaptic growth [81, 82]. Therefore, we propose that cAMP plays an important role in the action of ATX and MPH in ADHD and requires further attention, which is supported by several studies. Firstly, reduced expression levels of cAMP response element modulator (CREM) were found in an ADHD rat model [83]. Secondly, CREM mutant mice display ADHD-like behaviors such as increased levels of physical activity [84]. Thirdly, enhanced glutamate release and phosphorylation of cAMP response element binding protein (CREB) at serine 133 may be associated with attention deficit [85]. With respect to differences between the two drugs, we found that the expression of *Octbeta1R* was reduced within C15 (OL3) and C18 (MBKC_b) in the MPH-treated group, and may also inhibit cAMP production via inhibitory G_0α_ [22]. This is likely different from *Octbeta2R*, which plays a role in enabling OA receptor activity. Thus, we speculate the amount of cAMP may play an important role in the drug-induced results of MPH and ATX.

The expression level of *5-HT1B* was lower following the administration of both drugs, but *5-HT1A* was only expressed at a low level following MPH administration (Fig. 4C). *5-HT1B* is regarded as the modulator of drug reinforcement, stress sensitivity, mood, anxiety, and aggression. In addition, reduced *5-HT1B* auto-receptor activity may have an antidepressant-like effect [86]. Previous studies have shown that DA and 5-HT neurons can interact anomalously in ADHD at the soma, terminal, and distant levels [87]. Moreover, 5-HT regulates DA activity through its receptors 5-hydroxytryptamine receptor 1B (*HTR1B*) or 5-hydroxytryptamine receptor 2A (*HTR2A*), and their dysfunction can lead to problems in “5-HT-DA dynamics” resulting in ADHD symptoms [88, 89]. These preliminary data suggest an important role for the serotonin system in the development of ADHD. Moreover, studies in animal models of ADHD indicate intimate interplay between 5-HT and dopaminergic neurotransmission [60]. At the optimal dose, we observed a marked decrease in its expression, especially in the MPH group. Although human studies have not confirmed these associations, animal studies have found MPH to be a HTR1A agonist [90], and it is speculated that the activation of *5-HT1A* may play a partial role in MPH-mediated DA release in the brain.

The receptor genes for GABA and Glu belong to “Amino acid receptor genes”. Expression changes were observed within different cell types in the MPH-treated group; however, there were almost no significant differences in expression in the ATX-treated group, which may suggest that these receptors are targets of MPH but not ATX. Previous research has shown that ADHD may be related to insufficient responses of the GABAergic system in frontostriatal circuitry [91]; thus, we propose that inhibition of receptor genes such as *GABA-B-R1* may be a potential mechanism for the treatment of ADHD using MPH. Additionally, many receptor genes for Glu, such as *GluClalpha*, *GluRIA*, *GluRIB*, *mGluR*, and *KaiR1D*, respond to MPH, supporting its important role. The structure, function, and regulation of the Glu receptor (GluR) family have been extensively studied, and evidence supports the disruption of these mechanisms in psychiatric disorders including ADHD [92]. Imaging studies in children and adults with ADHD have revealed increased levels of Glu in the prefrontal cortex (PFC) and striatum [93]. Emerging evidence also suggests that psychostimulants target Glu receptors in the PFC neurons of monkeys and rats [11, 94]. Thus, a dysfunctional Glu system in the PFC may be a key contributor to ADHD phenotypes. These results suggest that the disrupted function of AMPARs in the PFC may cause the behavioral deficits in adolescent spontaneously hypertensive rats (SHR) and that enhancing PFC activity may be a successful treatment strategy for ADHD [95]. More importantly, experimental evidence shows that specific psychostimulants, such as *d*-amphetamine (AMP) and methamphetamine (MA), increase the levels of glutamatergic compounds in the human brain and that glutamatergic changes predict the extent and magnitude of subjective responses to these drugs [96]. There was also a reduction in the expression of “Acetylcholine receptor genes” following treatment with MPH, as shown in Fig. 4C. For example, *nAChRalpha6* and *nAChRalpha7* had lower expression levels in a variety of cell clusters, *nAChRalpha1* had a lower expression level in C22 (OL6), and *nAChbeta1* had a lower expression level in C18 (MKBC_b). This lower expression was not obvious in the ATX group, which also supports the notion that MPH is a broad inhibitor of amino acids and acetylcholine in the brain. Although there are no approved medications for ADHD that target nicotinic acetylcholine receptor (nAChR) function, results from many clinical trials have been reviewed, revealing that nicotinic drugs are typically well tolerated and present only mild-to-moderate side effects [97]. Generally, the number of differentially expressed receptor genes following MPH treatment is much higher than that following ATX treatment, which is consistent with the existing evidence that MPH is a much broader inhibitor. The patterns of receptor genes for different neurotransmitters support the view that MPH also affects the regulation of OA (NE), 5-HT, and Glu, and even other more general cellular processes [8, 9].

Neurotransmitter release requires the involvement of synaptic vesicles, and a variety of molecules and proteins play essential roles in mediating the binding and release of synaptic vesicles to neurotransmitters. The DEGs for “Neuronal and synaptic activity” and “Regulation of synaptic transmission” are summarized in Fig. 4C. The DEGs of the cell types in the MPH-treated group changed more broadly than those in the ATX-treated group. In addition, it must be emphasized that the expression level of *Hr38* changed in opposite directions in the MPH-treated and ATX-treated groups. *Hr38* is a *Drosophila* homolog of the mammalian Nr4a1/Nr4a2/Nr4a3 gene family, which is transcriptionally activated by *MEF2* in humans. Evidence shows that *Hr38* is also a downstream gene of *Mef2* in *Drosophila*, and alcohol activates *Mef2* to induce *Hr38*. An increased level of *Hr38* is associated with higher tolerance and increased preference for alcohol [98]. Moreover, knockdown of dopaminergic (*dMEF2*) neurons results in increased locomotor activity and reduced sleep, which is concordant with the human phenotype [28]. It should also be highlighted that *Snap25* was only responsive in the MPH-treated group. It is well known that synaptosome-associated protein of 25 kDa (SNAP25) is one of the critical proteins of the soluble N-ethylmaleimide-sensitive factor attachment protein receptor (SNARE) complex, which is essential for calcium-dependent exocytosis of synaptic vesicles. Dysfunction of the SNARE complex and related proteins is involved in neurological disorders and highlights their significant contribution to the pathology of various neurological disorders such as ADHD and epilepsy, and genetic and pharmacogenetic evidence suggests that they may also be important biological targets for these diseases [99]. Other genes including *Syn*, *Rim*, *nSyb* and *Sh* responded to MPH and ATX treatment, and existing evidence also supports their key roles in psychiatric disorders such as ADHD [100]. For example, Synapsin III (Syn III) is a neuronal phosphoprotein that regulates striatal dopaminergic neurotransmission in the adult brain, supporting the finding that MPH can directly interact with Syn III [101]. Synapsin III also regulates dopaminergic neuronal development in vertebrates [102].

In summary, in addition to DA-related genes, MPH and ATX also inhibit other receptor genes, such as those for OA/5-HT/GABA/Glu/Ach, to similar or differing degrees in *Drosophila* brain, and their patterns are summarized as follows. Firstly, the stimulant MPH induced a wider range of inhibitory effects than the non-stimulant ATX, and published research indicates that MPH regulates multiple pathways in *Drosophila* brain [8, 9]. MPH exhibits a larger inhibition of receptor genes than ATX, not only DA receptors but also others, and the expression response patterns of the two drugs are generally distinct. Some receptor genes share certain characteristics with one another, suggesting that they serve the same purpose in several pharmacological therapies. These findings provide the basic effects of psychotropic drugs at single-cell resolution in *Drosophila* brain. Inspiringly, a recent study mapped the transcriptome of the caudate nucleus and anterior cingulate cortex in post-mortem tissue from 60 individuals with and without ADHD, uncovering significant downregulation of neurotransmitter gene pathways, especially glutamatergic [58]. Specifically, glutamate receptor genes are enriched by DEGs in the caudate nucleus, DEGs in the anterior cingulate cortex (ACC) are involved in serotonin and GABA receptor activity, and a broad set of genes for neurotransmitter receptor activity is enriched by DEGs in both regions [58]. This transcriptomics evidence highlights corticostriatal neurotransmitter abnormalities in the pathogenesis of ADHD, especially receptor genes for different neurotransmitters, suggesting that our *Drosophila* drug discovery data are consistent with neurotransmitter results in post-mortem tissue of humans with ADHD and have the potential for large-scale drug screening. These are the most promising data for ADHD, including the target genes and their corresponding repurposing drugs; therefore, it is essential to translate these results into human orthologous genes and investigate more deeply.

### Subdivision of glial cells and their essential role in MPH and ATX effects

**Fig. 5:**
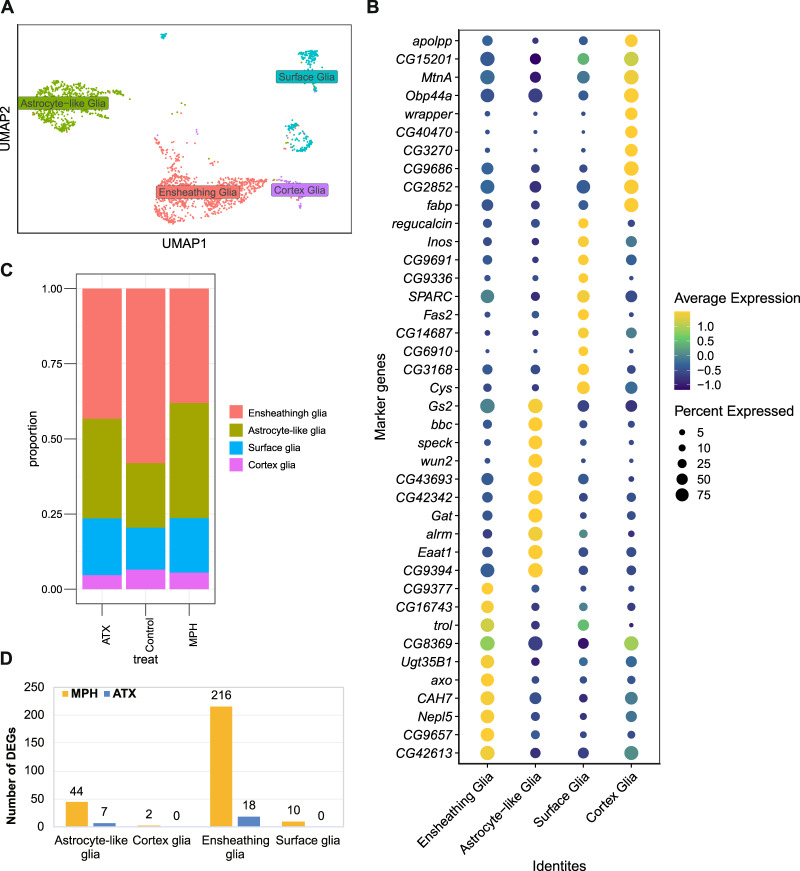
Subdivision of glial cells in adult *Drosophila* brain and their response to psychotropic drugs. **A** Re-clustering of the cells labeled in the dim plot (Fig. 2A). Only the subglial cells (ensheathing, astrocyte-like, surface, and cortex glia) are shown and analyzed. **B** Dot plot (40 genes in total) showing the top 10 genes with positive expression in each distinguished glial population (pairwise comparisons for genes expressed in >25% of cells in either cluster; Log2 FC > 0.25, MAST test with Bonferroni-adjusted *P*  ≤ 0.05). Dot diameter represents the fraction of cells expressing the gene (row value) in the cell cluster (column value), as shown in the scale. Color intensity represents the average normalized expression level. **C** The proportion of glial cell subtypes following each of the three treatments are displayed. **D** Statistics of the DEGs in different subglia. DEGs are mainly enriched in astrocyte-like and ensheathing glia.

As mentioned previously, glia possesses a large number of DEGs that can be used to address the potential effects of drug treatment. Moreover, glial cells are primarily important for sustaining and maintaining appropriate neuronal function. Emerging evidence indicates that distinct subtypes of glia play a crucial role in the control of neuronal development, apoptosis, metabolism, sleep, and other physiological activities in *Drosophila* [24]. There exist five types of glial cells in *Drosophila* including astrocyte-like, ensheathing, cortex, perineurial, and subperineurial glia, with the last two types belonging to surface glial cells. Identification of specific subtypes of glial cells is essential to explore their function in response to drugs in our data; therefore, we extracted cells from the Glial (C7) cluster, re-clustered them at a low resolution, and filtered the mixed neuronal cells. We identified four sub-clusters of glial cells: ensheathing, astrocyte-like, surface, and cortex glia, as shown in Fig. 5. All the subglial cells are labeled in the UMAP plot in Fig. 5A, and the top 10 genes with positive expression in each distinguished glial population are shown in the dot plot in Fig. 5B. Some of the top 10 genes consistent with published markers of glia were confirmed. Surface-associated glia are located in the outer adult brain and form the blood-brain-barrier (BBB) to help fruit flies detect their required nutrients [103]. Cortex glial cells constitute the layer below the surface cells and wrap around multiple neuronal cell bodies, participating in the metabolic support of neurons and providing nutrients [104]. Ensheathing and astrocyte-like glial cells belong to neuropil-associated glia and are located around the nerve bundle, forming a sheath structure. Ensheathing glial cells have been shown to remove degenerated axon fragments after brain injury [105]. Astrocyte-like glial cells play an important role in information transmission between neurons and glial cells, in addition to guaranteeing neurotransmitter homeostasis by expressing a set of specific transporter proteins such as excitatory amino acid transporter 1 (EAAT1) or GABA transporter (GAT) [106–108]. Previous studies have indicated that drugs can also induce a response in glial cells; for instance, esketamine alleviates cortical microglial activation, alters microglial number, and maintains morphological features in mice [109]. Moreover, MPH increases Glu uptake in chick cerebellum Bergmann glial cells [110], and different doses of MPH induce different glial cells (e.g., astrocytes, microglia) under ADHD or non-ADHD conditions in rats [111]. More direct evidence in humans shows that neuronal and glial cell numbers are altered in a cortical layer-specific manner in autism [112]. Overall, although there exists no direct evidence for the effect of MPH or ATX at the cellular level in the human brain, our results demonstrate that both drugs induced a different response in *Drosophila* brain, which needs to be addressed. More specifically, in comparison with the control group, the subtype proportions estimated based on the expression of marker genes changed in both the MPH-treated and ATX-treated groups (Fig. 5C): there were relatively increased surface and astrocyte-like glia but a relatively decreased proportion of ensheathing and cortex glia. These changes show that MPH and ATX play a specific role in regulating subglial cells; however, the underlying mechanism needs to be further analyzed.

The DEGs for each subglial cell type were calculated separately and the counts are shown in the bar graph in Fig. 5D. Astrocyte-like and ensheathing glia had strikingly apparent gene expression changes, with more than 10 DEGs in each group. Detailed results of the DEGs can be found in Supplementary Table 4. Subsequently, pathway analysis was conducted in subglial cells possessing more than 10 DEGs. Pathways for the DEGs in ensheathing glia following MPH treatment were enriched in various biological processes, such as responses to stimuli, rhythmic processes, locomotion, metabolic processes, and developmental processes, revealing key processes that respond to MPH treatment; however, no specific pathways were identified for DEGs following ATX treatment. For example, semaphorin 1a (*Sema1a*), which was differentially expressed following MPH treatment, encodes a transmembrane protein involved in the negative regulation of locomotion, and a previous study showed that glial cells overexpressing another family member, *Sema2a*, cause abnormal traveling of flies [113]. Additionally, *bendless* (ben) is expressed in ensheathing glial cells, which encodes an E2 ubiquitin-conjugating enzyme that plays essential roles in multiple processes such as synaptic growth and maturation, axon guidance, innate immunity, genomic integrity, tumor growth, apoptosis, and long-term memory. Furthermore, the pathways related to DEGs in astrocyte-like glial cells following MPH treatment were also enriched in metabolic processes, cellular processes, and responses to stimuli. Accordingly, MPH has been shown to activate astrocytes in limbic neuronal/glial co-cultures [114]. Differential expression of the excitatory amino acid transporter 1 (*Eaat1*) in astrocyte-like glial cells following MPH treatment participates in glia–neuron communication [115] and tightly regulates extracellular Glu levels to control neurotransmitter functions in locomotor behavior [108].

In summary, four subtypes of glial cells were identified and shown to have different functions in adult *Drosophila* brain. Genes and pathways responsive to MPH and ATX treatment indicate the key role of various subglial cells at the brain level, especially ensheathing and astrocyte-like glia. Moreover, human neurodevelopmental and neurodegenerative central nervous system diseases associated with glial dysfunction in *Drosophila* models have been reviewed and summarized [116], uncovering the contribution of glial cells to brain function and disease susceptibility.

#### Cytochrome P450 genes occupy only a small fraction of cells in adult Drosophila brain, most of which are expressed in glial cells

As mentioned previously, most DEGs between drug-treated and control cells are involved in metabolic processes; therefore, we also analyzed the cytochrome P450 (CYP) genes in our data. P450 enzymes are heme-thiolate proteins best known for their role as monooxygenases and are present in almost all living organisms. Insect CYP genes can be assigned to four different phylogenetically related “clans,” three named after the founding family in vertebrates (CYP3, CYP4, and CYP2 clans) and one named according to their subcellular location (mitochondrial CYP clan). We analyzed 87 genes from these groups in *Drosophila melanogaster* recorded in FlyBase and found that only a small proportion were expressed in our fly brain data (Supplementary Fig. 4). In addition, a single-nucleus RNA sequencing study in the adult *Drosophila* renal system reported the presence of CYP genes *Cyp6g1* and *Cyp12d1* [117] in tubules, which are known to detoxify insecticides. However, *Cyp6g1* was found within our fly brain data in very few cells, and no cells expressed *Cyp12d1*, indicating tissue differences in the expression of CYP genes in *Drosophila*.

The detected genes were primarily expressed in glial cells, but only occupied a small percentage of glia and were present at varying levels (Supplementary Fig. 4A); their distribution in subglial cells is shown in Supplementary Fig. 4B. Moreover, CYP genes were not identified as DEGs following drug treatment due to the small proportion of expressing cells in adult *Drosophila* brain (Supplementary Fig. 4C). Comparison of the expression levels of CYP genes in glial subtypes between the drug-treated and control groups revealed some related functions that should be noted. *Cyp6a2* [118] and *Cyp12a4* [119] are associated with insecticide resistance; *Cyp6a20* is thought to be related to aggressive behavior [120]; *Cyp28c1*, *Cyp311a1*, and *Cyp4d2* were shown to be lethal in an RNAi screen, and *Cyp4s3* was sublethal in that screen [121]; and *Cyp28a5* was expressed in different directions in different subglial cells and is related to monooxygenase activity, and has been reported to be induced by caffeine [122].

In general, CYP genes only occupied a small proportion of cells in adult *Drosophila* brain, which were mainly glia, and the functions of most of these genes are not well understood. We did not identify any significantly differentially expressed CYP genes involved in drug metabolism in adult *Drosophila* brain, which may be due to their limited percentage or tissue specificity.

#### Cell–cell communication analysis identified plausible interactions between monoaminergic neurons and glial cells

FlyPhoneDB [123] can effectively identify active ligands and receptors and predict cell–cell communication events between cell clusters in adult *Drosophila* brain. We analyzed the 28 major pathways separately in the three treatments (MPH, ATX, and control) and uncovered their cell–cell interaction pairs between the different cell types (Supplementary Fig. 5A). Notably, the Hippo, JAK/STAT, and Torso signaling pathways displayed no interactions within any cell clusters in our adult *Drosophila* brain data. Further, in most cases, glial cells were the major center of the cell communication network, especially in the EGFR, FGFR, Hedgehog, Insulin, Notch, TNF-α, and Toll signaling pathways, also suggesting its central role in adult *Drosophila* brain. In addition, monoaminergic cells showed strong connections with glial cells in the EGFR, FGFR, Insulin, Notch, and TGF-β signaling pathway. In particular, we found some differences in specific signaling pathways between the drug-treated and control groups; for example, the TNF-α signaling pathway connects monoaminergic neurons and glia following MPH treatment, while this connection is made through the *egr_wgn* ligand–receptor pair rather than in ATX-treated or control group (Supplementary Fig. 5B). It has been reported that another similar stimulant, methamphetamine, activates microglia by critically modulating astrocyte-derived TNF and Glu in adult mouse brain [124]. Additionally, the Toll signaling pathway is a major regulator of innate immunity in *Drosophila* and indicates more connections and different directions of expression following MPH or ATX treatment as compared with the control group. The FGFR signaling pathway highlights the importance of glia in the assembly and maintenance of neural circuits and the functions of FGF signaling in these processes [125]. Detailed results can be found in Supplementary Table 5. Despite the fact that the precise effects are unknown, these findings suggest a potential immunological response triggered by both drugs. A previous study has shown that astrocyte signaling and gliotransmitters represent the highly evolved integrative interface in brain communication that is coupled to slow modulatory signaling from multiple sources with fast synaptic transmission [126]. As mentioned previously, our results show the important role of glia in DA reuptake, metabolism and recycling (Fig. 4), and responsive genes, cells, and pathways following MPH and ATX treatment, indicating the key role of various subglial cells at the brain level, especially ensheathing and astrocyte-like glia (Fig. 5). Our cell–cell communication prediction results show that after MPH and ATX treatment, glial cells specifically interact with monoaminergic neurons through a variety of ligand–receptor pairings. We harbor the view that these connections participate in glia–neuron communication to regulate and control the neurotransmission elicited by MPH and ATX; nevertheless, it is necessary to confirm the specifics of these interactions in the future.

### Drug-responsive DEGs can be translated to human orthologs to generate drug potential

**Fig. 6:**
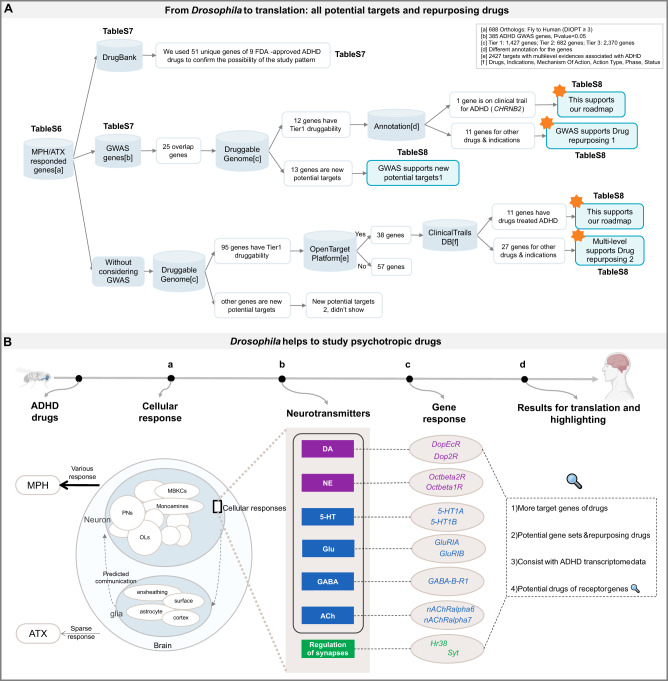
Translation of drug-responsive DEGs to humans and a summary of our research paradigm and results. **A** General workflow, datasets, and results for the translation of drug-responsive DEGs to humans. Starting with the human orthologs (DIOPT score $\ge$ 3) targeted by MPH and ATX, annotation information used for each dataset is summarized in the box in the upper right corner (a, b, c, …, f). Genes, drugs, and indications are presented in the corresponding supplementary tables shown in the figure. The datasets marked with orange asterisks are those that we wish to highlight. **B** *Drosophila* was employed to study psychotropic drugs, with ADHD drugs as an example. (a) Cellular response. Results contain both neurons and glial cells in *Drosophila* brain, and their predicted inter-communications. Circles represent different cell types; blank circles indicate a great number of neurons to discover in the future. MPH elicits various cellular responses, but there is a sparse pattern following ATX treatment. (b) Exploration of the neurotransmitter hypothesis. Exploration of the neurotransmitter hypothesis includes DA (major), OA (NE), 5-HT, Glu, GABA, and Ach, in addition to synaptic regulation. (c) Gene response. Gene responses together with their related neurotransmitter or pathway were analyzed, some of which are marked on the plot. (d) Results for translation and highlighting. Main results from our *Drosophila* pattern that are crucial and need to be emphasized: (1) We propose more target genes of drugs than previous studies; (2) These genes have great potential, and known drugs can be considered for repurposing; (3) Our receptor genes are consistent with the ADHD transcriptomics data; and (4) Results provide potential gene sets and repurposing drugs for ADHD, highlighting possible drugs for receptor genes. These findings confirm the reliability and validity of the psychotropic drug pattern in *Drosophila*.

As mentioned earlier, our results provide the most promising candidates and foundation for ADHD in *Drosophila* brain; therefore, it is essential to translate these data to human orthologous target genes and their corresponding repurposing drugs. More than 70% of human orthologs (DIOPT score $\ge$ 3) were found in cells following both MPH and ATX treatment (Supplementary Table 6), which provides raw drug-response mapping data that further contribute to the establishment of a theoretical basis in humans. Existing data were used to confirm the possibility of our pattern in *Drosophila*, and then prospective target genes and repurposing drugs were analyzed. A general workflow and the datasets used are shown in Fig. 6A.

#### Known mapping data of targets to drugs confirms the possibility of potential drug screening in Drosophila

After comparing the orthologs of the DEGs with the target genes of nine FDA-approved ADHD drugs recorded in DrugBank (Supplementary Table 7), overlaps were found for *DRD2* (fly: *Dop2R*), *HTR1A* (fly: *5-HT1A, 5-HT1B*), *SLC22A5* (fly: *CG7084*), *SLC22A4* (fly: *CG7084*), *ADRB2* (fly: *Octbeta2R*), *ADRA2A* (fly: *Octalpha2R*). These are targets of different psychotropic drugs including Amphetamine, Methylphenidate, Clonidine, Guanfacine, and Methamphetamine; for example, *DRD2* is the target gene of various drugs, among which is Amphetamine [127], but the pharmacological response is unknown. Additionally, *DRD2* is also associated with several neuropsychiatric disorders including ADHD, autism spectrum disorder (ASD), and bipolar disorder (BD) [128]. These results strongly support our pipeline to efficiently identify potential drug targets for ADHD.

#### Overlaps between drug-responsive genes, GWAS candidate genes, and druggable genes

The relationship between susceptibility genes and drug target genes in psychiatric disorders and ADHD is a controversial topic. Previous studies have shown little association between drug target genes and ADHD susceptibility genes [39, 129], which may be due to the limited number of known ADHD drug target genes and published GWAS candidate genes. Thus, after obtaining the drug-responsive genes for *Drosophila*, we reconsidered the relationship among them. More crucially, focusing on genes known to encode druggable proteins will help to translate the results to humans. Beyond the first estimated core pharmacological principles [37], an increasing number of potentially druggable genes have been defined by Finan et al. as set of 4479 divided into 3 tiers based on druggability levels [38]. Several studies have been conducted using these druggable genes to explore more targets or repurposing drugs for different conditions including ADHD [39], nootropics [42], Parkinson’s disease [43], and schizophrenia [44]. Some putative genes targeted by existing drugs potentially available for repurposing have been identified through this approach; therefore, drug repurposing is further embraced in the framework of our *Drosophila* single-cell brain study to uncover more possibilities.

We demonstrated the relationship between the unique orthologs of the DEGs following treatment with MPH or ATX (688 orthologs with DIOPT score $\ge$ 3 in Supplementary Table 6) and the genome-wide association genes for ADHD (385 genes reported previously [39] and shown in Supplementary Table 7). Drugs with genetic evidence to support their target in relation to the indication are more likely to be successfully approved than drugs lacking such evidence [130]. Large-scale GWAS studies have uncovered new drug targets for the treatment of psychiatric disorders [131–133]. For ADHD, 25 overlapping genes were found after expanding the drug target genes to the *Drosophila* drug-responsive genes, among which 12 genes have Tier 1 druggability and 13 genes with Tier 2/3 can be treated as new potential targets. Detailed information can be found in Supplementary Table 8. In particular, Tier 1 contains targets of approved small molecules, biotherapeutic drugs, and drugs in clinical trials [38]. Annotation of these 12 druggable genes using the Open Target Platform [134] (https://platform.opentargets.org/), which provides multi-level evidence, showed that drugs targeting *CHRNB2*, such as AZD1446 (interacts with cholinergic neurons), have been in clinical trials for ADHD (e.g., NCT01012375). Other genes and their 41 mapped drugs and indications providing evidence for drug repurposing can be found in Supplementary Table 8.

#### More prospective target genes and repurposing drugs are supported by existing evidence

Without relying on any particular hypothesis, we also examined the prospective capabilities of these orthologs and presented them according to their levels of druggability. In total, 232 druggable DEGs were identified in *Drosophila* following MPH treatment and 105 druggable DEGs were found following ATX treatment. Among these, 95 unique genes belong to Tier 1. Next, we searched all published targets that have multi-level evidence associated with ADHD (2427 genes) in the Open Target Platform to support our unique Tier 1 druggable genes, and 38 genes were retained after filtering. Moreover, information related to the target genes, such as the corresponding drugs, indications, mechanism of action (MoA), action type, and clinical phase and status were added to aid exploration of the clinical trials database (https://clinicaltrials.gov). After combining this information, 11 genes corresponding to various drugs related to ADHD that were in different clinical stages (drugs approved by the FDA or in on-going investigation) were found. For example, GUANFACINE and CLONIDINE are approved by the FDA and used clinically; others (more than 20 drugs), such as LIDOCAINE, BUSPIRONE, MK-8777, and MOLINDONE, have been investigated at different clinical stages of ADHD. Details of the clinical trials are summarized in Supplementary Table 8. These results confirm the efficacy of our *Drosophila* drug screening system and provide a foundation for future large-scale extension. The hope is that these drugs (mapped to 27 genes) that respond to other indications have not yet been tested for ADHD and may be repurposed in the future. Multiple lines of evidence suggest that these 27 genes have the most robust evidence for ADHD-associated risk. These potential drugs and their corresponding indications are listed in Supplementary Table 8. Genes belonging to Tier 2 and Tier 3 were treated as additional potential targets and are not shown due to their vast number.

#### Drug set enrichment analysis confirms the effectiveness of our approach

To confirm that the drug repurposing results found using our approach are indeed relevant to ADHD treatment, we conducted drug set enrichment analysis similarly to the well-known gene set enrichment analysis (GSEA) [135], with the drug set replacing the gene set. We conducted a hypergeometric test [135, 136] to calculate the *P-*value for our drug repurposing results; the lower the *P-*value, the better the performance of drug repurposing. Our results display *P* = 2.10E-4, indicating effective performance of our drug repurposing framework. More details are listed in the Methods section.

Here, we summarize and emphasize a number of these prospective new targets and drugs for repurposing with different levels of evidence as shown in Supplementary Table 8. Drug and target information can also be retrieved through our web tool ADHDrug (http://adhdrug.cibr.ac.cn/). These data are richer and more comprehensive than previously published data for ADHD. Ongoing or completed examples at different clinical stages of ADHD give us the confidence to elucidate new treatments for ADHD using drug repurposing approaches. To conclude, we present a framework for the exploration of potential druggable genes in *Drosophila* using two ADHD drugs and the possibilities for drug repurposing (Supplementary Table 8) as potential novel avenues for ADHD treatment. Our findings add to the knowledge of known ADHD drugs at the single-cell level and expand our exploration of ADHD-related drug repurposing, which may provide interventions at the multi-evidence level of the disease.

## Discussion

Since it is difficult to access human samples, particularly the brains of children or adults with ADHD, it continues to be almost impossible to study drug effects at single-cell resolution. Therefore, we selected *Drosophila melanogaster* as our model due to advantages such as speed, high-throughput, and low cost. Adult *Drosophila* brain cell types have been thoroughly analyzed, resulting in a high-resolution single-cell atlas [31–33]. In the present study, we conducted single-cell transcriptomics analysis of six adult male *Drosophila* brain samples containing gene expression changes under three conditions: MPH-treated, ATX-treated, and control. Our research adds valuable insights and information regarding the following topics: (1) “hyperactivity-like” behavior induced by MPH or ATX treatment were automatically tracked and analyzed by EasyFlyTracker in adult male *Drosophila*; (2) the distribution of cell types and genes responsive to the psychiatric drugs MPH and ATX are shown at the brain level; (3) possible biological functions of cell types and gene expression status following the response to drug treatment are shown, especially the “neurotransmitter regulation hypothesis” related to ADHD; (4) results for translating data from *Drosophila* to humans and drug repurposing potentials are provided. The key to this study is the use of fruit flies to elucidate the molecular mechanism of the two most widely used ADHD drugs (MPH and ATX) at the single-cell level. A summary of our research paradigm and results can be found in Fig. 6B, including cellular response, neurotransmitter hypothesis exploration, gene response, and data for the translation from *Drosophila* to humans. On one hand, different cell types reacted differently to the drugs, revealing heterogeneity in cellular responses within the brain, which is largely ignored but extremely important for the development of existing drug response theory.

Specifically, both the stimulant MPH and the non-stimulant ATX induced higher locomotor activities at optimal drug doses, and the cellular responses to MPH and ATX were widespread in both glia and neurons, including MBKCs, Monoamines, PNs, and OLs, as shown in Fig. 6B(a). However, the effects of the stimulant MPH were more widespread and intense than those of the non-stimulant ATX. Additionally, the results for MPH were in accordance with previous widespread responses induced by acute cocaine exposure [35] in male adult *Drosophila*. The major responsive cell types shared only a limited number of pathways in *Drosophila* brain, indicating that cell types perform different functions following MPH and ATX treatment. We mainly focused on the known biological functions corresponding to drugs in humans, including monoamines, neurotransmitters, and glial cells. Unknown clusters and their corresponding functions are ignored here. We found that limited cells simultaneously release both excitatory and inhibitory neurotransmitters, which is consistent with the scRNASEQ results in larval brain [32] and midbrain [33]. Nevertheless, this phenomenon was different from previous findings indicating that GABAergic inhibitory neurons in *Drosophila* do not express either of the excitatory transmitters (Glu or ACh) [137]. We propose that this discrepancy may be due to timepoint differences or the capture of multiple cells, which needs to be explored with other possibilities in the future. Moreover, our findings highlight two possible differing mechanisms for the change in the proportions of neurons and glial cells induced by these drugs: neurotransmitter switching and cell differentiation. We found that the effects of MPH and ATX induced changes in the cell type proportions of GABAergic neurons according to the expression levels of key genes. MPH also elicited a significant response in monoaminergic neurons, indicating that the stimulant MPH had a stronger effect on the regulation of monoamines. Furthermore, both drugs only affected the gene expression level of a small number of cells. In the future, it may be possible to directly detect dynamic neurotransmitter levels in vivo using a genetically encoded sensor for 5-HT or DA, as previously reported [138, 139]. Our results also address the contributions of glial cells to the effect of psychotropic drugs in the brain, especially ensheathing and astrocyte-like glia. A previous study of the effects of cocaine on *Drosophila* brain also suggested that altered expression of *Eaat1* in astrocytes may play a role in cocaine-induced locomotor effects [35]. Glia is likely to contribute to the metabolism of drugs, which deserves further study. Overall, our findings highlight the importance of the exploration of diverse brain cellular responses to psychotropic drugs. Although we cannot directly map cell types between flies and humans, the various cell types in *Drosophila* brain respond differently to MPH and ATX, further explaining the diversity of drug responses and the importance of precise therapy.

In particular, transcriptional effects of both drugs related to the “neurotransmitter regulation hypothesis” were fully analyzed. Explorations included DA, NE, 5-HT, Glu, GABA, and ACh as well as synaptic regulation, as shown in Fig. 6B (b). We harbor the view that more targets of MPH and ATX than simply *DAT* can be mined for the following reasons. Some examples are shown in Fig. 6B (c). Firstly, with respect to genes involved in DA metabolism and signaling pathways, the DA receptor genes *Dop2R* and *DopEcR* were differentially expressed between the MPH-treated and control groups, whereas there were no differences between the ATX-treated and control groups. Our findings support the former view that various genes are crucial in ADHD, in addition to certain regulatory genes such as *Syt1*, *Sytalpha*, *Syt7*, and *Ih*. As addressed previously, certain reasonable factors including drug dose, the differing structure of DAT between humans and *Drosophila*, and other targets, prevent us from visualizing the pattern of *DAT* inhibition after drug treatment. Additionally, MPH and ATX also inhibited receptor genes for other neurotransmitters. Even more importantly, the results for the drug effects in *Drosophila* brain are consistent with the transcriptomics data for the caudate nucleus and anterior cingulate cortex in post-mortem tissue from 60 patients with and without ADHD. Differentially expressed Glu receptor genes were enriched in the caudate nucleus, and DEGs in the anterior cingulate cortex were involved in 5-HT and GABA receptor activity [58]. Corticostriatal neurotransmitter abnormalities in the pathogenesis of ADHD were highlighted, especially genes for different neurotransmitter receptors. The overlapping data indicate the reliability and validity of large-scale psychotropic drug screening at single-cell resolution. We regard these receptor genes as the most significant findings, and known targeted compounds can be considered as repurposing drugs with the most potential.

The human orthologs of drug-responsive genes provide original drug-response mapping data, which in turn contribute to the establishment of a theoretical basis for translation to humans. Here, we summarize our main *Drosophila* brain data that are likely important in humans (Fig. 6B (d)). Firstly, it may help to identify more susceptibility genes for ADHD. Previous studies in *Drosophila* have shown that target homologous genes, such as *GARNL3*, *SLC6A3*, *LPHN3*, *NF1*, *MEF2C*, and *TRAPPC9*, cause ADHD-like behaviors [25–28]. Here, we uncovered *Hr38*, a homolog of *NR4A2*, which is transcriptionally activated by *MEF2* in humans. *NR4A2* alterations have been linked to DA-associated brain disorders such as Parkinson’s disease and schizophrenia. A previous study reported that *NR4A2* deficiency is associated with ADHD-like phenotypes in mice [140]. Moreover, knockdown of dopaminergic (*dMEF2*) neurons results in increased locomotor activity and reduced sleep, which is concordant with the human phenotype [28]. Recently, *MEF2C* gene variations have been associated with ADHD in the Chinese Han population [141]. Secondly, more targets and their corresponding drugs can be explored. ATX is a famous successful repurposed drug, previously for Parkinson’s disease, which was approved for ADHD in November 2002 [40]. Thus, providing the most potential targets and known drugs is the prospective application of our study. Certain genes with known drugs already applied to ADHD are good examples to validate our pattern. Drugs that respond to other indications (which map to 27 genes) have not yet been tested clinically for ADHD, and these drugs (Supplementary Table 8) may be repurposed in the future. In particular, drugs targeting receptor genes for neurotransmitters can be considered as repurposing drugs with the most potential. We regard these receptor genes as the most significant findings; therefore, these genes and their known drugs are displayed in Supplementary Table 8. Examples include ARBACLOFEN (*GABBR1*), POMAGLUMETAD METHIONIL (*GRM3*), and TOPIRAMATE (*GRIK1*). For instance, POMAGLUMETAD METHIONIL (*GRM3*) is a metabotropic glutamate receptor 3 agonist that has been tested for SZ (https://clinicaltrials.gov/ct2/show/NCT01452919) and TOPIRAMATE (*GRIK1*) has been used for BD (NCT00240721, NCT00035230, NCT00237289), cognitive impairment (NCT02884050), and PD (NCT00296959). Although target genes or drugs are not further prioritized in this paper due to the complexity of the issue, our future work is an essential part of the avenue to translational medicine; therefore, we will continue to investigate as soon as possible. Thirdly, although some genes, such as *MEF2C* and *NR4A2*, are not targets of known drugs, they still play important roles as both susceptibility and drug-responsive genes. Additionally, *MEF2C* is also a susceptibility gene of AD identified by the GWAS of European ancestry [142]. Recently, variations in the *MEF2C* gene have been associated with ADHD in the Chinese Han population [141], indicating its key role. In conclusion, we have summarized and highlighted some of the potential new targets with differing degrees of evidence, and reusable relevant information can be found through our web tool ADHDrug (http://adhdrug.cibr.ac.cn). The combination of *Drosophila* and single-cell expression approaches make it possible to explore the effects of psychotropic drugs rapidly at a high throughput and low cost. It is a fact that healthy animals are different from diseases models, which means that our candidate repurposing drug lists are not necessarily related to ADHD treatment; however, our framework is still valid since it is supported by drug set enrichment analysis and can be used as a rapid screening tool. In addition, disease models can also be different from ADHD patients. In our further studies, we will incorporate diseases models to continuously enrich our understanding of ADHD drug targets. Moreover, we can expand our conditions to directly explore dose effects. Since the current study focused on the optimal drug dose and a single exposure of male flies, there is also a need to consider gender or developmental effects in the future. Even as we improve our understanding of the response of *Drosophila* brain to the two common drugs MPH and ATX, targeted validation will still be needed in the future. We have already provided more possibilities for repurposing drugs and a set of candidates with the most potential; however, there remains a need for valid statistical models and further analytical techniques to predict drug priorities. We recognize the gap between the current results and clinical applications, and the above-mentioned improvements can be achieved when more resources are available.

## Conclusion

Here, we propose an innovative research paradigm for ADHD treatment using *Drosophila*, explore potential targets of ADHD, and provide a candidate list for drug repurposing. We strongly believe that our *Drosophila* research paradigm, although not immediately applicable to the clinic, can be prospectively applied to further research the pathogenesis of ADHD or other psychiatric disorders and the use of cell type-specific marker genes, target screening, and drug repurposing in the future.

## Methods

### *Drosophila* breeding

Wild-type (WT) *w*^*1118*^ *Drosophila melanogaster* was obtained from the FangJing Company, and the population was maintained at 25 °C under a 12 h:12 h light/dark photoperiod. All flies were reared on standard *Drosophila* medium (corn, sugar, yeast, agar) in a 25 °C climate chamber with ~60% relative humidity and a 12 h:12 h light/dark cycle. Flies were 3- to 5-day-old adult males (after eclosion) at the time of behavioral activity experimentation.

### Behavioral activity assay

The behavioral activity assay included different parts, as shown in Fig. 1.

#### Modified capillary feeder (CAFE) assay

After 15 h of starvation, flies were exposed to one of the three treatments (control, MPH, or ATX) (Sigma–Aldrich China, Shanghai) using the modified capillary feeder (CAFE) assay [45] for approximately 24 h to visually control food intake (Fig. 1A). Five (~3 days old) males were transferred to feeding vials containing two 5-µL capillary tubes extending down into the vial. The feeding vials were topped with an oil layer and placed within a tightly sealed container at high humidity (~90%) to minimize evaporation.

#### Dose–response curves for MPH/ATX

After selecting a dose according to the literature [10], drugs were tested at four or five different doses (for ATX: 0.25 mg mL^-1^, 0.5 mg mL^-1^, 1 mg mL^-1^, and 2 mg mL^-1^; for MPH: 0.25 mg mL^-1^, 0.5 mg mL^-1^, 1 mg mL^-1^, 1.5 mg mL^-1^, and 2 mg mL^-1^) to find the inflection point of the dose–response curves. The average distance traveled (mm) was calculated for each drug dose by removing the control effects of that day: (Distance_(Drug dose A)_-Distance_(Control)_)/Distance_(Control)_. The Kruskal test was used to calculate the difference between any two doses, and boxplots were created using Python. Concentrations of 0.25 mg mL^-1^ ATX (Supplementary Fig. 1A) and 1.5 mg mL^-1^ MPH (Supplementary Fig. 1B) had the strongest effect and were chosen for subsequent experiments. The control treatment consisted of 5% sucrose (SUC) and yeast solution (with 5% blue food dye, Sigma–Aldrich China, Shanghai); the methylphenidate (MPH) treatment contained 5% SUC and yeast solution (with 5% blue food dye) and 1.5 mg mL^-1^ MPH; and the atomoxetine (ATX) treatment contained 5% SUC and yeast solution (with 5% blue food dye) and 0.25 mg mL^-1^ ATX.

#### Locomotor activity analysis

Flies were relocated from the CAFE assay to our customized environments for video recording and analysis (Fig. 1B, C). The setup and software details can be found in our previous study evaluating EasyFlyTracker [29]. We placed one fly in each hole and engaged in simultaneous tracking (24 flies/treatment; 72 flies in total) of the customized activity chambers. The activity plate was placed on top of a light box and enclosed within a separate room to minimize external disturbance. After the flies had adapted to the recording environment (lights were turned on, usually from 09:00 to 10:00 am), video was recorded using a camera at a resolution of 1280 × 720 with 30 frames per second (fps) for 2.5 h, and EasyFlyTracker was subsequently used to track and analyze the locomotor activities of the flies in different treatment groups from the saved videos. Two experiments were performed separately on July 12th (replicate 1) and August 10th (replicate 2) 2021. The Kruskal–Wallis test (with Bonferroni correction) was used to calculate significant changes in the short-term distances between the MPH-treated or ATX-treated group and the control group throughout the 2.5-h videos. EasyFlyTracker also created angle-change plots (Supplementary Fig. 1C) and heatmaps (Supplementary Fig. 1D) for the different treatments to display more details of the behavioral activities of the fruit flies. Pearson *r* values for the angle-change and locomotor activities in both the drug-treated and control groups are shown as a scatter plot in Supplementary Fig. 1C.

### Brain dissection, dissociation, and creation of a single-cell suspension

At the end of the behavioral experiment, the fruit flies were anesthetized with CO_2_ immediately after removal of dead or inactive flies, transferred to a glass dissection plate containing cold PBS, and then the flies’ heads were dissected using clean tweezers under a high-definition Motic (SMZ-168-BLED) stereomicroscope. The duration between the end of the behavioral test and the start of dissection was maintained the shortest possible for each batch. We used a dissociation protocol modified from Croset et al. [33] and Davie et al. [31]; the detailed protocol can be found in *Supplementary Materials*. We collected 6 samples of 20 brains from flies exposed to MPH, ATX, or control treatment, with two biological replicates per treatment. Samples with a count $>$500 live cells/μL were used to prepare the sequencing library.

### Library preparation and sequencing

scRNASEQ libraries were prepared using the Chromium Next GEM Single Cell 3’ Reagent Kit v3.1 (10X Genomics) based on the manufacturer’s instructions (User Guide). The library conversion kit App-A of MGIEasy was used to generate the libraries. Double-end sequencing of the final libraries was performed on an MGISEQ-2000 platform according to the manufacturer’s instructions. The assessment of PCR product purity and library quality was performed on the Agilent Bioanalyzer 2100 system.

### scRNASEQ data processing

#### FASTQ generation, demultiplexing, and alignment

The Basecall software was used to automatically convert CAL files from the sequence run folder to demultiplexed FASTQ files. The reference genome was built (indexed using the mkref pipeline) based on the *Drosophila melanogaster* reference named “dmel-all-r6.39.gtf.gz” from the FlyBase database (ftp.flybase.net) and aligned using the count pipeline within Cell Ranger v6.0.1. The sequencing and alignment summary is provided in Supplementary Table 1.

#### Preprocessing, integration, and cell type clustering

Raw expression counts for each sample from the CellRanger pipeline (default parameters) were imported and analyzed using the Seurat v4.0.3 package in R v4.1.0. A few criteria were commonly used to remove low-quality (multiple, broken, empty) cells [143]. Genes expressed in less than five cells and cells with less than 200 or greater than 4000 RNA features were filtered out, and the mitochondrial gene percentage was controlled to be less than 15% (percent.mt$<$15). Doublets were also predicted and removed by DoubletFinder [46]. After filtering, a total of 82,917 cells remained for subsequent analysis. A filtered gene-barcode matrix of all samples was normalized and integrated using the scTransform pipeline [144]. After integration, PCA was performed and the UMAP was created with the top 15 principal components to visualize the cells. Meanwhile, unsupervised clustering was performed on the PCA-reduced data using the FindClusters function for clustering analysis with Seurat v4.0.3. The resolution was set to 0.1 to identify the primary cell type clusters.

Cluster marker genes were identified using the FindAllMarker function (min.pct equals 0.25; logfc.threshold equals 0.25; only.pos equals TRUE; assay equals SCT; slot equals data; test.use equals MAST). Canonical markers and the top genes were combined to identify cell types, as shown in Supplementary Table 2. Most major cell types from different tissues, such as neurons and glia, can be distinguished from one another using unique markers. However, certain cell types can be further divided into functionally distinct subtypes, which typically requires the use of multiple markers rather than a single unique marker [145]. The top 10 genes with positive expression for each cluster were extracted and used for cell type characterization.

#### Differentially expressed gene and pathway analysis

Differentially expressed gene analysis was performed for each cluster to evaluate the effect of the drugs separately after combining MPH-exposed, ATX-exposed, and control samples. The Pearson residuals output from the sctransform pipeline was used as input for the differentially expressed gene (DEG) calculation [144]. The MAST [146] algorithm was used as the testing methodology in the FindMarkers function (test.use equals “MAST”; assay equals “SCT”; slot equals “data”) for each cluster for the DEG calculation.

Pathway enrichment analysis was globally conducted based on all DEGs between the MPH-treated or ATX-treated groups and the control group, and then clusters with a sufficient number of DEGs were subjected to pathway enrichment analysis using the online software Metascape (http://metascape.org/) [147]. Results were automatically generated by Metascape using default parameters (gene annotations automatically retrieved from the latest version of the database are shown. All genes in the genome were used as the enrichment background). Terms with *P* < 0.01, a minimum count of 3, and an enrichment factor >1.5 (the enrichment factor is the ratio between the observed counts and the counts expected by chance) were collected and grouped into clusters based on their membership similarities. The top 20 clusters with their representative enriched terms (one per cluster) are provided. *P-*values were calculated based on the cumulative hypergeometric distribution. “Log10(q)” is the multi-test adjusted *p-value* in log base 10, which was calculated using the Benjamini–Hochberg procedure). Pathways with a multi-test adjusted *P* $<$ 0.05 were considered statistically enriched.

#### Re-clustering of specific primary clusters

After cells were clustered and defined at the primary level, re-clustering was used to identify subclusters within certain specific clusters, including monoaminergic neurons and glia. Similar analysis was performed for each primary cluster according to the following steps: (1) extracting cells from the primary cluster of the integrated cells; (2) performing another PCA and choosing suitable PCs; (3) specifying a resolution and visualizing the clusters in a UMAP plot; (4) assigning and annotating cell identities after analyzing differentially expressed marker genes as described previously; (5) performing subsequent analysis based on the DEGs of specific subclusters. Detailed parameters of the different primary clusters are shown below.

##### Monoamines

In this case, 15 PCs with a resolution of 0.3 were selected to generate a UMAP plot and new clusters. Known markers of neurons releasing 5-HT, tyramine (TA), octopamine (OA), and DA in *Drosophila* allowed us to identify the sub-clusters corresponding to each of these cell types. *Dopa decarboxylase (Ddc)* labels 5-HT and DA neurons; *Serotonin transporter (SerT)* and *Tryptophan hydroxylase (Trh)* mark 5-HT neurons; *pale* (*ple*; tyrosine hydroxylase) and DAT label DA neurons; *Tyrosine decarboxylase 2 (Tdc2)* marks TA and OA neurons; and *Tyramine β-hydroxylase (Tbh)* labels OA neurons. Cells respond to monoamines using the expression of *Vmat*, which only occupied a small proportion of all cells in our adult male brain datasets (Supplementary Fig. 3A). One cluster called Monoamines (C20) clearly expressed *Vmat*, as shown in the Feature Plot, at a markedly higher level than that in other cell clusters such as C0, C5, and C7 (Supplementary Fig. 3A). In addition to *Vmat*, marker genes such as *ple* and *DAT* are also mainly expressed in the Monoamines (C20) rather than in other clusters such as C0, C5, and C7 (Supplementary Fig. 3B); therefore, we only selected this portion to perform another PCA and UMAP analysis on cells from the Monoamine (C20) cluster (Supplementary Fig. 3C). There were no subclusters assigned after considering the top marker and classical genes. Thus, we regarded Monoamines (C20) as our research target representing dopaminergic neurons.

##### Glial cells

In this case, 20 PCs with a resolution of 0.04 were selected to generate a UMAP plot and new clusters. Cluster names were assigned after considering the top marker and classical genes. Only subglial cells were considered and subsequently analyzed.

#### CYP gene analysis in adult Drosophila brain

Insect CYP genes can be assigned to four different phylogenetically related ‘clans’ named after the founding family in vertebrates (CYP3, CYP4, CYP2 clans) or their subcellular location (mitochondrial CYP clan). A total of 87 CYP genes in *Drosophila melanogaster* recorded on FlyBase were chosen, and their distribution in the primary clusters was analyzed. Only enriched glial cells were selected, and the effects of these CYP genes in subglial cells after drug treatment were evaluated.

#### Cell–cell communication analysis

Cell–cell communication analysis was performed using FlyPhoneDB [123]. Previously processed gene expression matrix and cell cluster information for the three treatments were used separately as the input for analysis. Ligand–receptor interaction scores and specificity were calculated. Cellular communications at the signaling pathway level were visualized using circle plots. The interactions of ligand–receptor pairs between two cell types were visualized by dot plots, with L-R interaction scores and specificity in the right panel. Heatmaps show the expression of the core components in the signaling pathway in all the brain cell types.

### Potential gene analysis: drug repurposing

We used scRNASEQ approaches in *Drosophila* to improve our understanding of the response of two commonly used drugs, MPH and ATX, in the brain. While not ready for immediate clinical use, we provide more exploration of target identification and drug repurposing opportunities using the following approach. The general steps of data analysis are comprised of three main components: (1) Human ortholog identification of drug-responsive genes; (2) Collection of different datasets; and (3) Gene to drug and disease annotations.

#### Human ortholog identification of drug-responsive genes

Human orthologs were obtained using the DRSC Integrative Ortholog Prediction Tool v8 [148]. Only genes with a DIOPT score $\ge$ 3 were considered human orthologs in subsequent analysis and are shown in Supplementary Table 6.

#### Collection of different datasets

##### Target genes of FDA-approved ADHD drugs

Nine drugs were selected according to their “Drug Description” containing “ADHD” in the DrugBank (https://go.drugbank.com/) database. Target enzyme and transporter genes were collected from the DrugBank of these nine drugs as a set of “ADHD drug target genes” and are shown in Supplementary Table 7.

##### GWAS genes of ADHD

A total of 385 genes found to have *P* < 0.05 in a previous study [39] were marked as GWAS genes of ADHD and are shown in Supplementary Table 7.

##### Druggable genome data

Druggable genes were defined by Finan et al. as a set of 4479 genes and were divided into 3 tiers based on druggability levels [38]. Tier 1 (1427 genes) contains targets of approved small molecules, biotherapeutic drugs, and those in clinical trials; Tier 2 (682 genes) encodes targets with a high sequence similarity (over ≥75% of the sequence) to Tier 1 proteins or those targeted by small drug-like molecules; and Tier 3 (2370 genes) contains genes encoding secreted and extracellular proteins, proteins with more distant similarity to approved drug targets, and members of key druggable gene families not already included in Tiers 1 or 2 (GPCRs, nuclear hormone receptors, ion channels, kinases, and phosphodiesterases) [38]. Annotations are shown in Supplementary Table 6.

##### Multiple evidence-based data

Targets with multi-level evidence associated with ADHD in the Open Target Platform (https://platform.opentargets.org/) were downloaded, which included 2427 genes (released data: 2022.6) [134]. All these genes include annotations from the ClinicalTrails.gov (https://clinicaltrials.gov) database, such as the corresponding drugs, indications, mechanism of action (MoA), action type, clinical phase, and status.

#### Gene to drug and disease annotations

All the above datasets and online tools were prepared for the annotation and filtering of genes at different levels.

##### GWAS support genes

Drugs with genetic evidence to support their target in relation to the indication are more likely to be successfully approved than drugs without such evidence [130]. Large-scale GWAS studies have discovered new drug targets for the treatment of major psychiatric disorders [131–133]; thus, only genes with GWAS evidence were retained.

##### Genes chosen without consideration of the GWAS hypothesis

To elucidate more possibilities, we also summarized the multi-level evidence supporting druggable genes and drugs for repurposing.

#### Drug set enrichment analysis

To verify that the drug repurposing results found by our approach are indeed relevant to ADHD treatment, we conducted drug set enrichment analysis similarly to the well-known gene set enrichment analysis (GSEA) [135], with the drug set replacing the gene set. The background drug set contains a total of $N$ drugs available for selection, from which the drug repurposing approach selected $n$ drugs. In addition, $M$ represents the number of drugs in the background drug set as well as those already approved for the treatment of ADHD. Among these $M$ drugs, $m$ drugs were found using our drug repurposing framework.

According to the description above, we used sampling without replacement to model this process, which will generate a probability mass function of hypergeometric distribution for $m$ drugs identified by our repurposing framework. Subsequently, we conducted a hypergeometric test [135, 136] to calculate the *P-value* for our drug repurposing results, the formula for which is as follows:

$$P=\mathop{\sum }\limits_{i=m}^{M}\frac{{C}_{M}^{i}{C}_{N-M}^{n-i}}{{C}_{N}^{n}}$$

The formula calculates the probability of finding the result that our drug set has greater than or equal to $m$ drugs overlapping with $M$ currently approved drugs to treat human ADHD. We suppose that the lower the *P-value*, the better the performance of drug repurposing. In our approach, the background drug set was taken from the ChEMBL [149] database (release version: CHEMBL30) containing 12,854 drugs and compounds, from which our approach selected 196 drugs. Therefore, we set $N$ = 12,854 and $n$ = 196. To confirm the drug set for ADHD treatment, we firstly selected approved drugs from the DrugBank (Supplementary Table 7). Next, we manually retained only those related to the above selected drugs from the DrugBank. Finally, we selected 20 drugs (Supplementary Table 7) to construct the ADHD drug set, of which 4 overlapped with our repurposing results. Therefore, we set $M$ = 20 and $m$ = 4 and calculated the *P-*value using the formula shown above.

### Web tool construction

We developed a web tool (http://adhdrug.cibr.ac.cn/) based on Shiny to better visualize and mine the datasets. Users can query the expression and statistics of genes in each cell type, and search the marker genes of all cell types or DEGs between the drug-treated and control groups. Users can plot the genes using different formats, such as FeaturePlot, VlnPlot, and DotPlot. Drug and target information can also be retrieved.

### Supplementary information

Supplementary Information Text

Supplementary Table 1

Supplementary Table 2

Supplementary Table 3

Supplementary Table 4

Supplementary Table 5

Supplementary Table 6

Supplementary Table 7

Supplementary Table 8

Supplementary Figure 1

Supplementary Figure 2

Supplementary Figure 3

Supplementary Figure 4

Supplementary Figure 5

## Data Availability

The raw sequence data reported in this paper have been deposited in the Genome Sequence Archive [150] at the National Genomics Data Center [151], China National Center for Bioinformation / Beijing Institute of Genomics, Chinese Academy of Sciences (GSA: CRA010939) and are publicly accessible at https://ngdc.cncb.ac.cn/gsa. The R code used to perform Seurat-based analysis and the Python code used to perform drug set enrichment analysis are available at GitHub https://github.com/Susuqu/scRNAseq_flydrug to ensure the replicability and reproducibility of these results.
