# Supplementary material for: The effects of methylphenidate and atomoxetine on Drosophila brain at single-cell resolution and potential drug repurposing for ADHD treatment
Source: Mol Psychiatry. 2023 Nov 13;29(1):165–85. doi: 10.1038/s41380-023-02314-6 (PMC11078728; doi:10.1038/s41380-023-02314-6)
Supplement: Supplementary file 1 — Supplementary Information Text [file 41380_2023_2314_MOESM1_ESM.docx]

**Supplementary Information for**

**The effects of methylphenidate and atomoxetine on *Drosophila* brain at single-cell resolution and potential drug repurposing for ADHD treatment**

**Susu Qu^1,2*^, Xiangyu Zhou^2^, Zhicheng Wang^2^, Yi Wei^2^, Han Zhou^2^, Xinshuang Zhang^2^, Qingjie Zhu^2^, Yanmin Wang^2^, Quanjun Yang^3^, Likun Jiang^4^, Yuan Ma^2^, Yuan Gao^2^, Lei Kong^5^, Li Zhang^2*^**

^1^Academy for Advanced Interdisciplinary Studies, Peking University, Beijing, China

^2^Chinese Institute for Brain Research, Beijing, China

^3^Department of Pharmacy, Shanghai Jiao Tong University Affiliated Sixth People’s Hospital, Shanghai, China

^4^Department of Computer Science, Xiamen University, Xiamen, China

^5^Center for Bioinformatics, State Key Laboratory of Protein and Plant Gene Research, School of Life Sciences, Peking University, Beijing, China

***Correspondence:**

Li Zhang

[zhangli@cibr.ac.cn](mailto:zhangli@cibr.ac.cn)

Susu Qu

[qususu@cibr.ac.cn](mailto:qususu@cibr.ac.cn)

**This file includes:**

Supplementary Materials

SI References

Legends for Supplementary Figures S1–S5

Legends for Supplementary Tables S1–S8

**Supplementary Materials**

**Protocol for brain dissection and dissociation to create a single-cell suspension**

After conducting the behavioral tests in adult male flies three days after eclosion, we began to dissect as soon as possible. Each batch consisted of three samples (with 20 brains per sample) that had been exposed to MPH, ATX, or control treatment. The dissection and dissociation protocol was adapted from ^1^, ^2^, and ^3^. Cold DPBS (sterile, 1×, without calcium and magnesium) was used to dissect the brains. Forceps were used to crush each of the 20 brains, which were then placed in a 0.5-mL tube as one sample. The dissection dish was cleaned with 200 $\mu L$ DPBS, and all the washes were combined with the sample. The samples were centrifuged at 800 × g and 4°C for 5 min, after which the supernatant was removed. Subsequently, 50$\mu$L 3 mg mL^-1^ dispase I (Sigma, D4818) and 75$\mu$L collagenase I (Invitrogen) were added and incubated for 10 min. Then, 125$\mu$L trypsin (0.05% Trypsin-EDTA, Invitrogen) was added and the samples were placed in the shaker and allowed to digest for 15 min at 37°C and 1000 rpm, being gently pipetted up and down every 5 min to mix thoroughly. Samples were subsequently centrifuged at 800 × g and 4°C for 5 min and the supernatant was discarded. Cells were resuspended in 150$\mu$L 0.04% BSA (Sigma) and transferred to a new 0.5-mL tube through a pre-wet 10-$\mu$m sterile pluriStrainer®. After washing the initial tubes twice with 0.04% BSA, the washes were filtered and mixed with the samples. Samples were centrifuged at 800 × g and 4°C for 5 min, the supernatant was removed, and the cells were resuspended in 400 $\mu$L 0.04% BSA. A 9-$\mu$L aliquot of each cell suspension was then added to a 0.2-mL tube and incubated for 10 min, after which 1 $\mu$L AO/PI dye was added to each sample. Live cells were counted using a LUNA-FL Dual Fluorescence Cell Counter. The samples were used for subsequent experiments at a count of > 500 live cells/$\mu$L.

**References**

1. Baker BM, Mokashi SS, Shankar V, Hatfield JS, Hannah RC, Mackay TFC *et al.* The Drosophila brain on cocaine at single-cell resolution. *Genome Res* 2021; **31**(10)**:** 1927-1937.

2. Croset V, Treiber CD, Waddell S. Cellular diversity in the Drosophila midbrain revealed by single-cell transcriptomics. *Elife* 2018; **7**.

3. Davie K, Janssens J, Koldere D, De Waegeneer M, Pech U, Kreft L *et al.* A Single-Cell Transcriptome Atlas of the Aging Drosophila Brain. *Cell* 2018; **174**(4)**:** 982-998 e920.

4. Hegvik TA, Waloen K, Pandey SK, Faraone SV, Haavik J, Zayats T. Druggable genome in attention deficit/hyperactivity disorder and its co-morbid conditions. New avenues for treatment. *Mol Psychiatry* 2021; **26**(8)**:** 4004-4015.

**Legends for Supplementary Figures S1–S5**

**Supplementary Figure 1. Optimal dose curve and other behavioral activities for the different treatments.**

The results of dose testing are shown in subfigure A and B for ATX and MPH, respectively. In general, drugs were tested at four or five different doses (for ATX: 0.25 mg mL^-1^, 0.5 mg mL^-1^, 1 mg mL^-1^, and 2 mg mL^-1^; for MPH: 0.25 mg mL^-1^, 0.5 mg mL^-1^, 1 mg mL^-1^, 1.5 mg mL^-1^, and 2 mg mL^-1^). The horizontal axis represents the different treatments at different doses (mg/mL), and the longitudinal axis shows the average distance traveled (mm) per fly in a 10-min time period. The average distance traveled (mm) was calculated for each drug dose by removing the control effects of that day: (Distance_(Drug dose A)_-Distance_(Control)_)/Distance_(Control)_. The Kruskal test was used to calculate the difference between any two doses, and boxplots were created using Python (ns: 0.05 < *P* $\leq$1; *: 1.00e-02 *< P* $\leq$5.00e-02; **: 0.001$<$*P* $\leq$0.01; ***: 0.0001 $<$*P* $\leq$0.001; ****: *P*$\leq$ 0.0001). (A) Optimal dose curve following the administration of ATX in *Drosophila*. (B) Optimal dose curve following the administration of MPH in *Drosophila*. As shown in the plot, 0.25 mg mL^-1^ ATX (in subfigure A) and 1.5 mg mL^-1^ MPH (in subfigure B) had the strongest effect and were chosen for subsequent experiments. (C) EasyFlyTracker illustrated the angle change of the fruit flies. (D) Movement plots of the different treatments displaying more details of the activities of the fruit flies.

**Supplementary Figure 2. Full plot of the biological pathways in certain cell types following MPH or ATX treatment by Metascape.**

Pathway analysis of selected neuronal clusters (C14, C18, and C20) by Metascape without filtering. Color bar represents the -log(p-value) of the pathway calculated using Metascape.

**Supplementary Figure 3. Subdivision of monoaminergic neurons.**

(A) Cells were classified based on Vesicular Monoamine Transporter (*Vmat*) labeling of one cluster from **Figure 2A** (cells in indigo, highlighted with arrowheads and circles). (B) Top marker genes of C20 from **Figure 2A**, clearly showing *Vmat* and *DAT*. (C) Re-clustering of the cells labeled in A showed no clear new cell types.

**Supplementary Figure 4. Expression of cytochrome P450 genes in adult *Drosophila* brain.**

(A) DotPlot of the 87 P450 genes in our *Drosophila* brain data. Only some were found and visualized in a small number of glial cells. (B) Each of the P450 genes in (A) were visualized in sub glial cells. (C) Each of the P450 genes in sub glial cells under different treatments were visualized by DotPlot.

**Supplementary Figure 5. Cell–cell communication analysis of different signaling pathways in adult *Drosophila* brain.**

(A) Circle plots of 13 signaling pathways in the *Drosophila* brain following the three treatments (MPH, ATX, and control). Each of the 28 cell clusters is displayed in a different color. The predicted interaction between two clusters is indicated by a colored curve. The thickness of the curve indicates the strength of the interaction. The full list of predicted ligand–receptor pair genes can be found in **Supplementary Table 5**. (B) The TNF-α signaling pathway is used as an example to explain the cell–cell communication differences between the drug and control groups. Circle plots show the communications between each cell cluster. Dot plots show the significant interactions between the ligand *egr* and its receptor *wgn* on monoaminergic neurons and glia. The legend “score” shows a dot plot of the interaction score and specificity of ligand–receptor pairs between one cell cluster and another. Heatmap patterns of the MPH-treated, ATX-treated, and control showing the activity of TNF-α signaling core components in the entire brain.

**Legends for Supplementary Tables S1–S8**

**Supplementary Table 1. Summary of the cell number and other statistics for each sample.**

Detailed statistics for the basic summary, sequencing quality, mapping rates, and estimated cell number for each sample are summarized.

**Supplementary Table 2. Summary of the canonical markers and top 10 marker genes used to annotate the clusters.**

The top 10 marker genes with positive expression in each cluster were extracted and summarized. Cluster annotation with colors is labeled for each line of the gene, which is in accordance with that used in **Figure 2A**. Neurons and glia were distinguished from one another using the known marker genes *elav* and *repo*. Other cell types typically require the use of multiple markers rather than a single unique marker.

**Supplementary Table 3.** **Full results of the general biological pathways of the shared common DEGs following MPH and ATX treatment analyzed by Metascape.**

These results were automatically generated by Metascape using default parameters. Gene annotations automatically retrieved from the latest version of the database are shown. All genes in the genome were used as the enrichment background. Terms with *P* < 0.01, a minimum count of 3, and an enrichment factor > 1.5 (the enrichment factor is the ratio between the observed counts and the counts expected by chance) were collected and grouped into clusters based on their membership similarities. The top 20 clusters with their representative enriched terms (one per cluster) are provided. *P*-values were calculated based on the cumulative hypergeometric distribution. "Log10(q)" is the multiple hypothesis testing adjusted *P-value* in log base 10, which was calculated using the Benjamini–Hochberg procedure.

**Supplementary Table 4. The full list of DEGs for each** **subglial cell type.**

DEGs following treatment with MPH and ATX for each subglial cell types are shown.

**Supplementary Table 5. The full list of predicted ligand/receptor pair genes for cell–cell communication following different treatments.**

**Supplementary Table 6. Summary of drug-response DEGs and their human orthologs and druggability levels.**

There are 694 and 248 unique DEGs following MPH and ATX treatment, respectively. Among the MPH-related DEGs, 561 (80.84%) have at least one human ortholog; and among the ATX-related DEGs, 196 (79.03%) have at least one human ortholog. Only human orthologs with a DIOPT score of 3 or greater are considered. Druggable gene annotations are shown.

**Supplementary Table 7. Summary of known ADHD drug targets and GWAS genes from previous studies.**

Target genes of 9 FDA-approved ADHD drugs are provided. All recordings are extracted from the DrugBank database with information for drug targets genes, enzymes and transporter genes. A total of 385 GWAS genes of ADHD with *P* $<$ 0.05 in previous study^4^ are also saved in the table.

**Supplementary Table 8. Summary of different levels of evidence for genes and repurposed drugs translated from *Drosophila* brain.**

Details regarding the different levels of evidence are summarized in different sheets. 1) “GWAS support genes”: ADHD GWAS data support 25 overlapping genes, including new potential targets and druggable genes. Their corresponding known drugs and indications are stored in the sheet named “GwasSupportDrugRepurposing1-OTP.” 2) Without considering the GWAS hypothesis, all published targets with multi-level evidence associated with ADHD help to filter genes and overlapping genes that are druggable are stored in the sheet named “MultiLevelDrugRepurposing2.” 3) Among the list of all repurposed drugs, some ADHD-related drugs are already in clinical use and others have been studied at different clinical trial stages. All these records are stored in the sheet named “ADHD clinical trials.”
