# Supplementary figures and images for: The effects of methylphenidate and atomoxetine on Drosophila brain at single-cell resolution and potential drug repurposing for ADHD treatment
Source: Mol Psychiatry. 2023 Nov 13;29(1):165–85. doi: 10.1038/s41380-023-02314-6 (PMC11078728; doi:10.1038/s41380-023-02314-6)

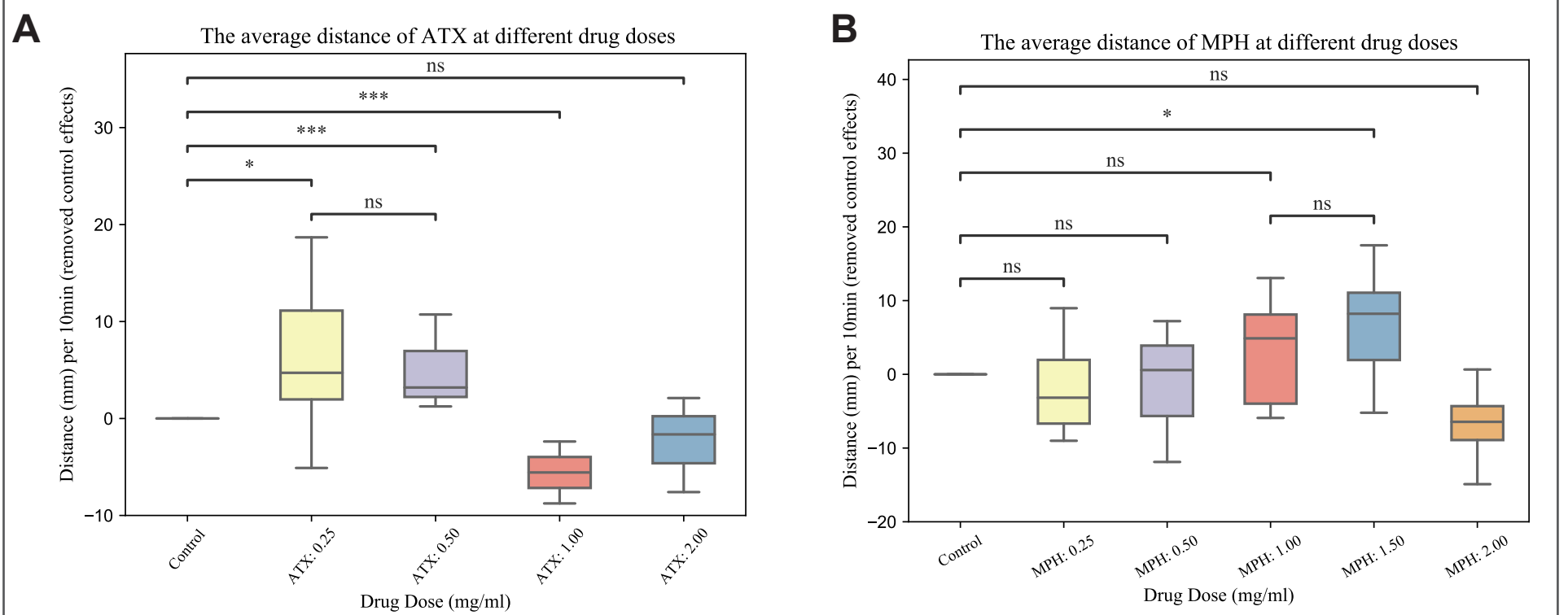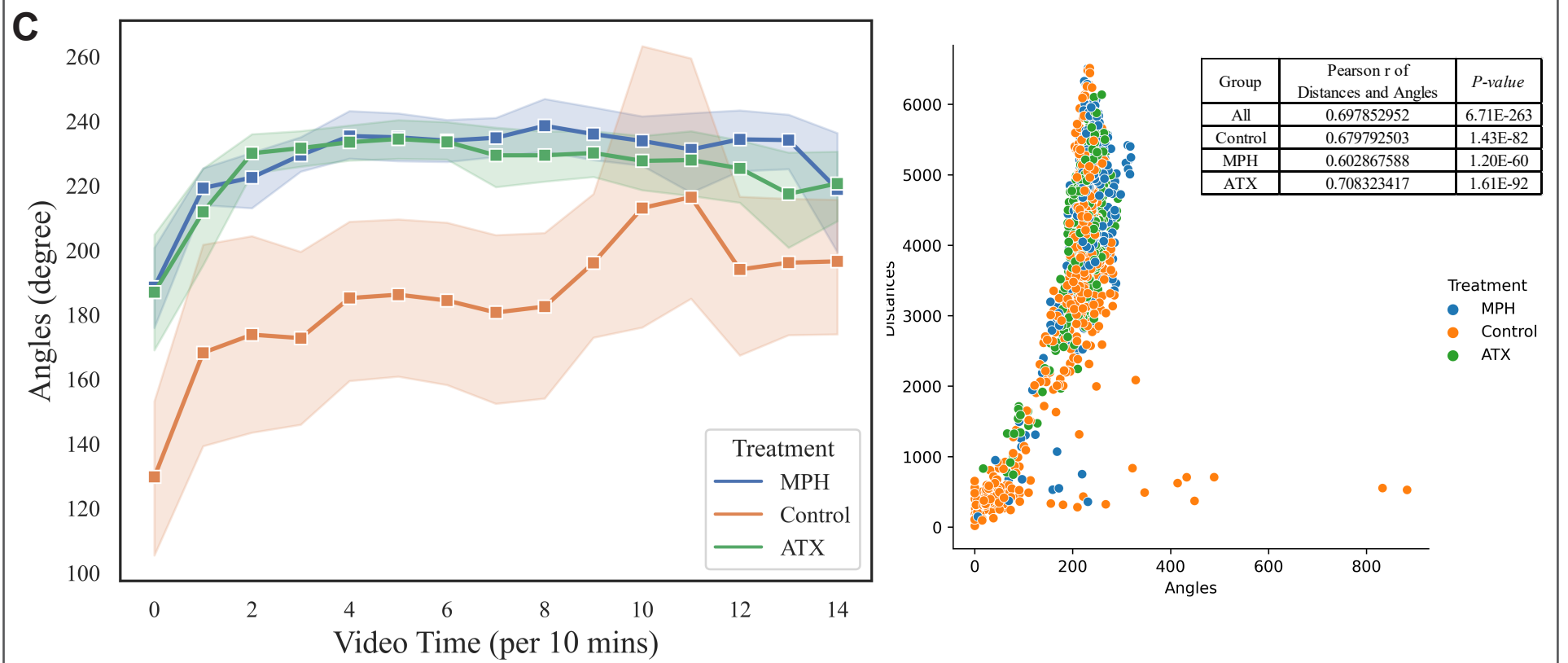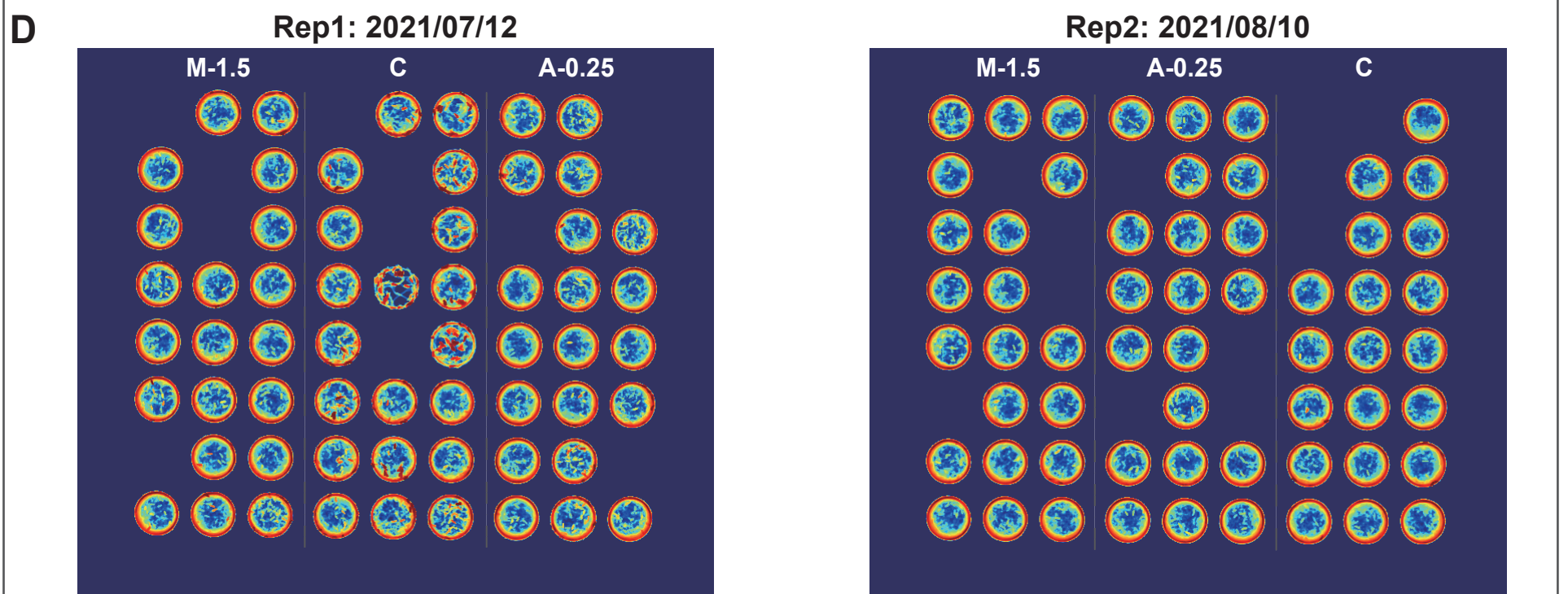

Supplement: Supplementary file 10 — Supplementary Figure 1 [file 41380_2023_2314_MOESM10_ESM.pdf]

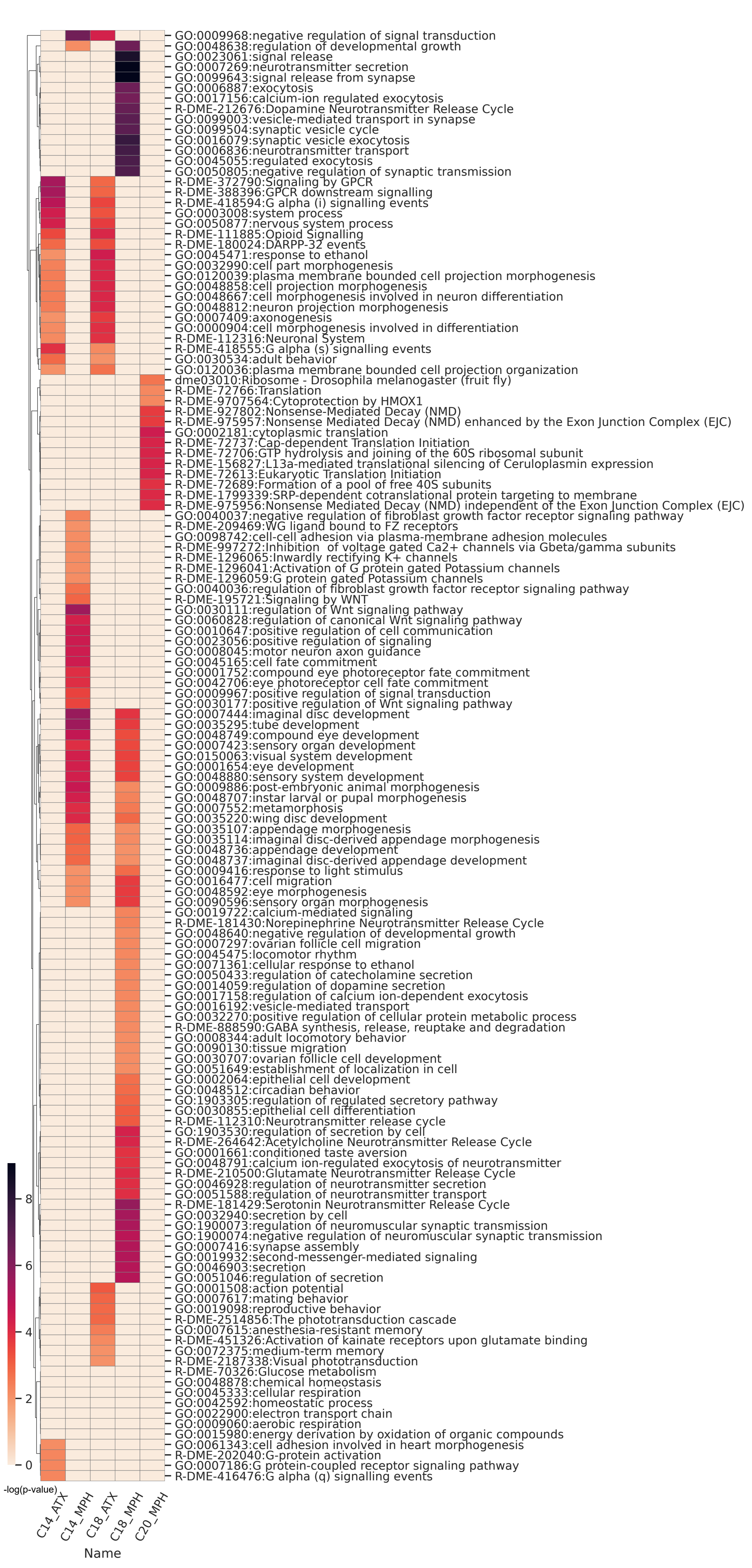

Supplement: Supplementary file 11 — Supplementary Figure 2 [file 41380_2023_2314_MOESM11_ESM.pdf]

A

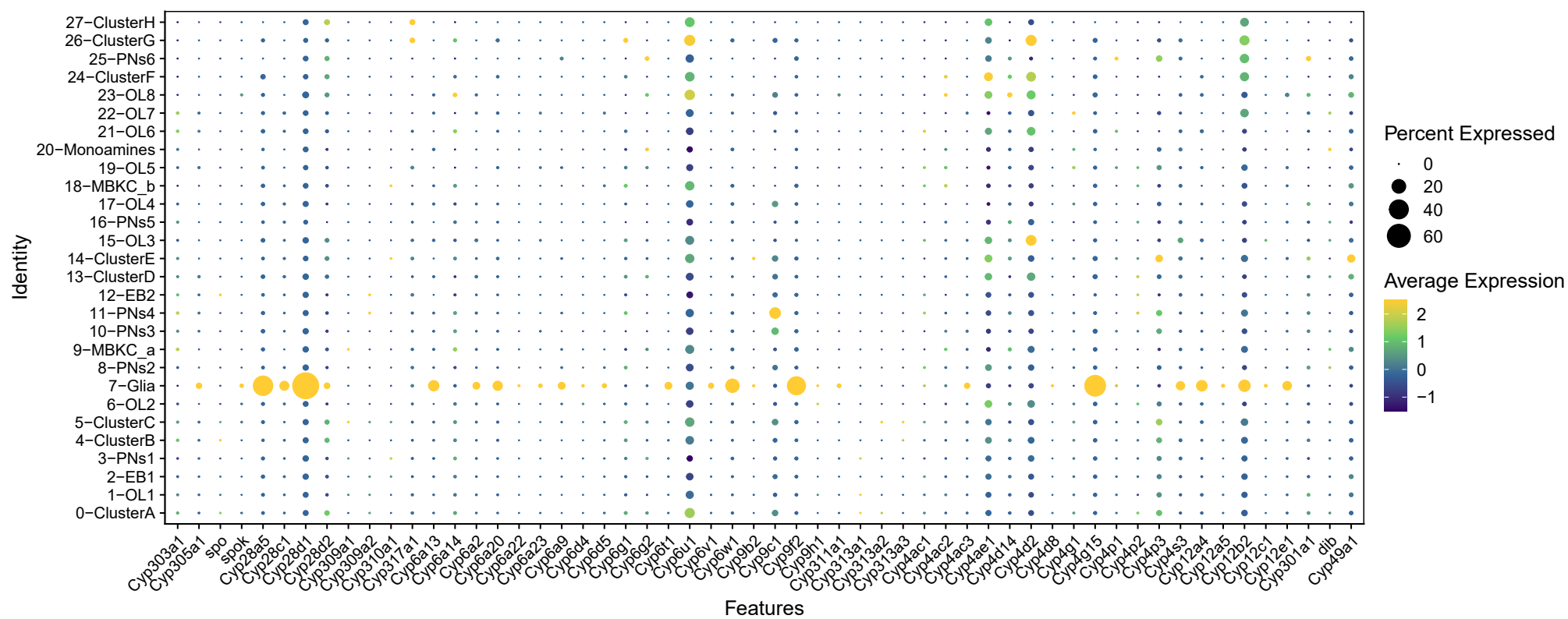

B

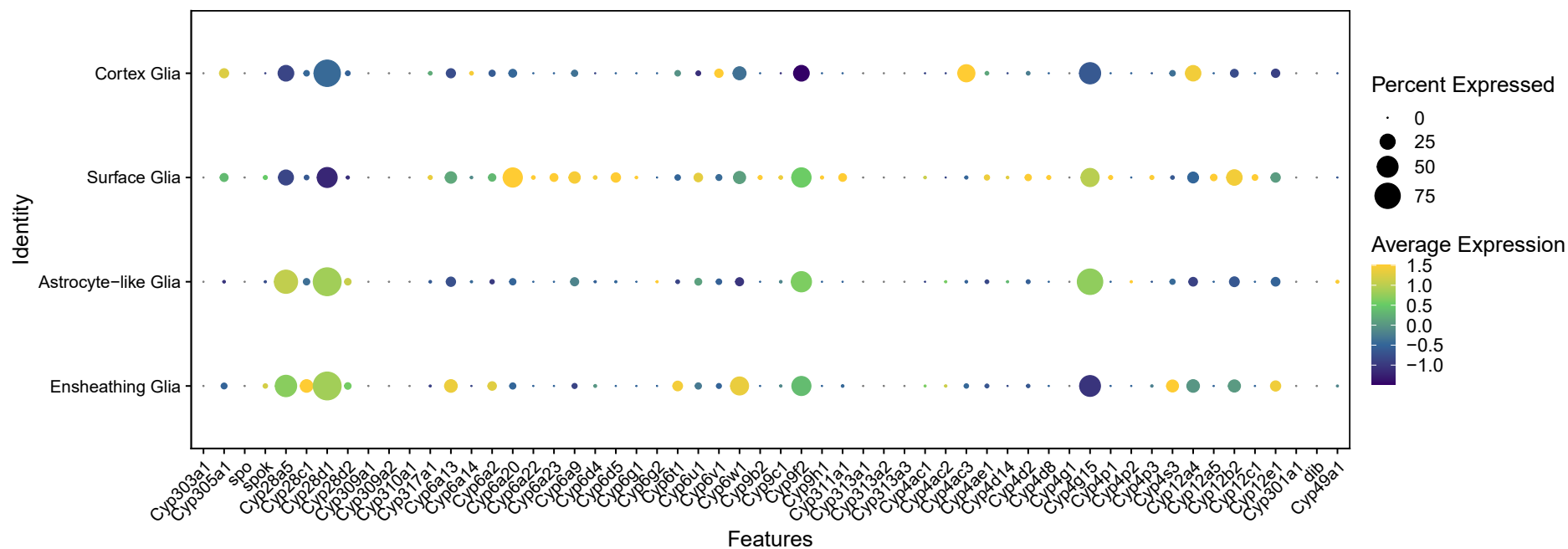

C

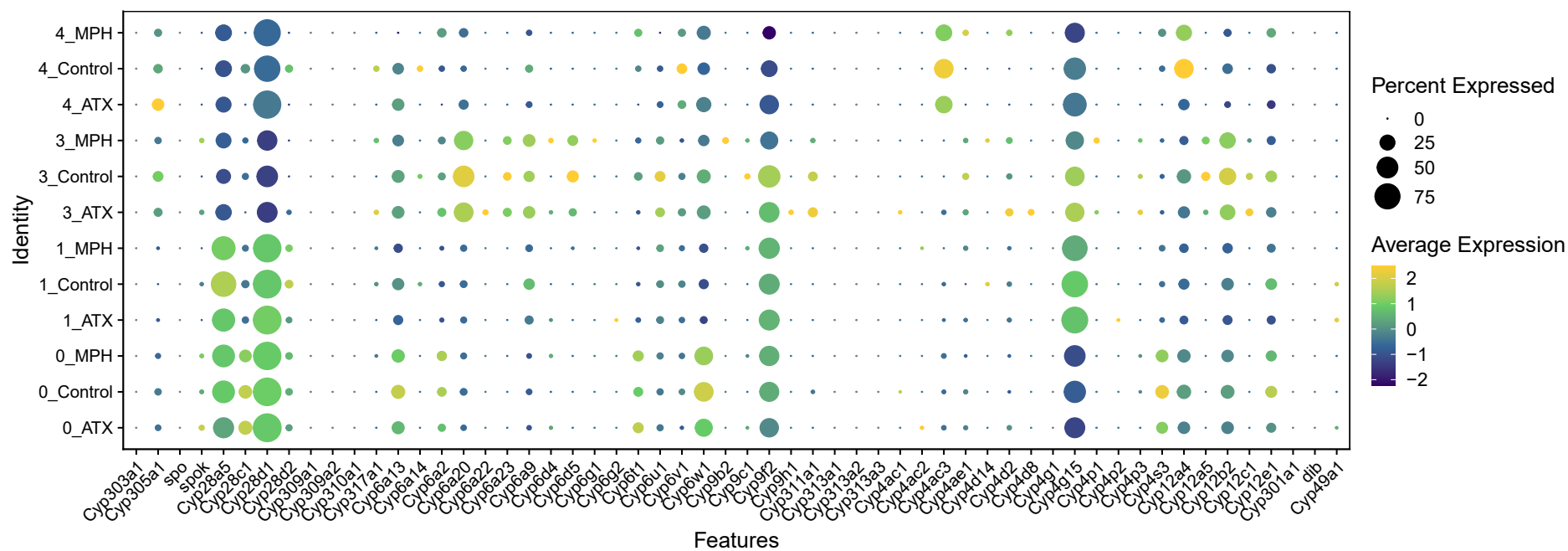

Supplement: Supplementary file 13 — Supplementary Figure 4 [file 41380_2023_2314_MOESM13_ESM.pdf]

A

MPH

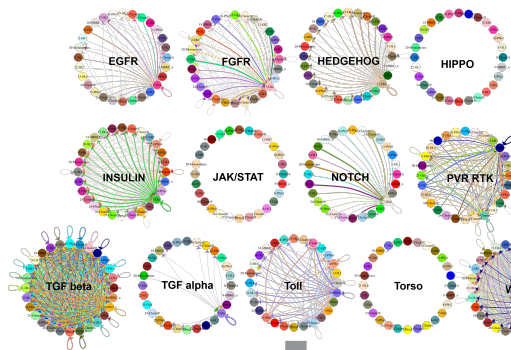

ATX

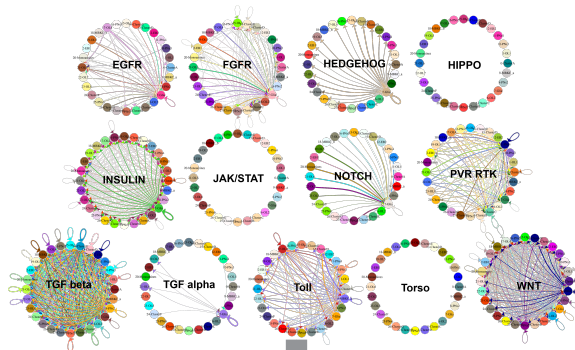

Control

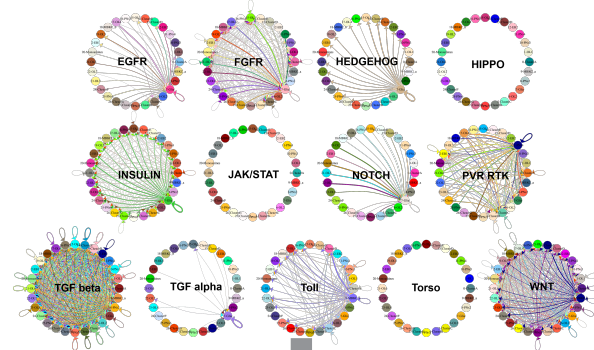

B

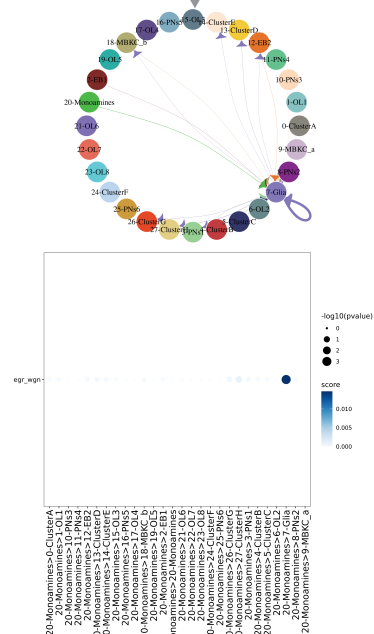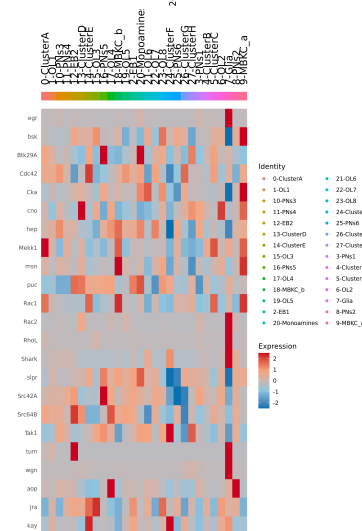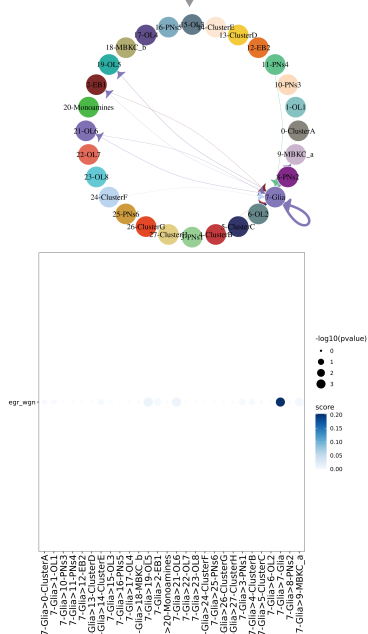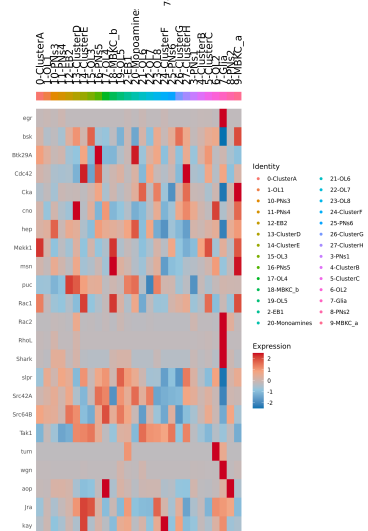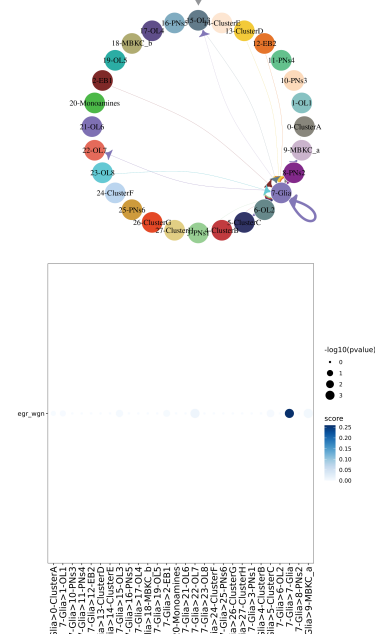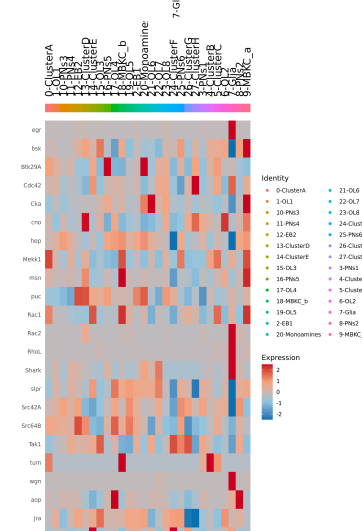

TNF alpha signaling pathway

Supplement: Supplementary file 14 — Supplementary Figure 5 [file 41380_2023_2314_MOESM14_ESM.pdf]
