# Supplementary material for: The effects of methylphenidate and atomoxetine on Drosophila brain at single-cell resolution and potential drug repurposing for ADHD treatment
Source: Mol Psychiatry. 2023 Nov 13;29(1):165–85. doi: 10.1038/s41380-023-02314-6 (PMC11078728; doi:10.1038/s41380-023-02314-6)

A

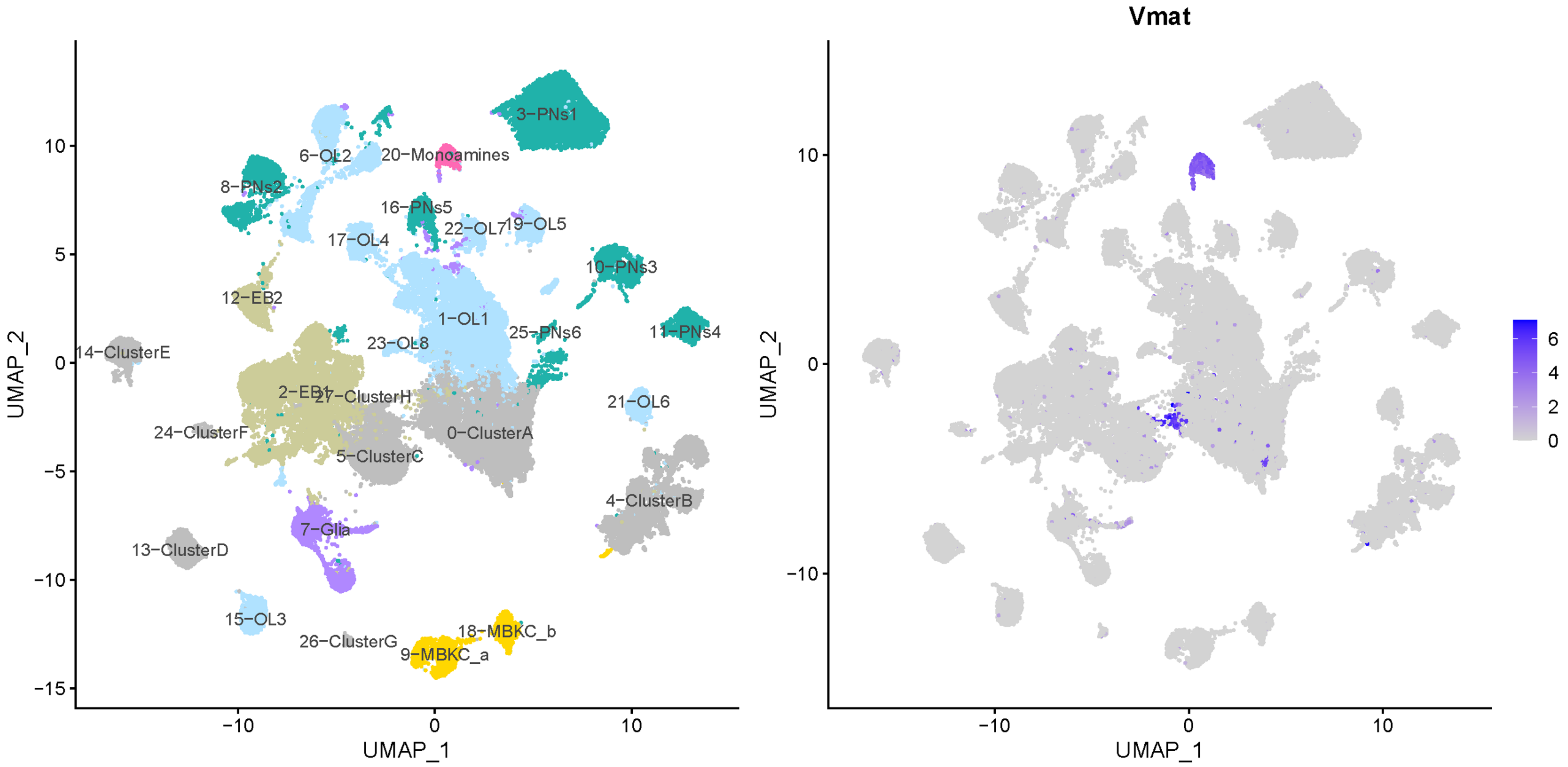

B

p_val	avg_log2FC	pct.1	pct.2	p_val_adj	cluster	gene-sct-mast
0	5.726031	0.995	0.02	0	20	*Vmat*
0	5.232617	0.976	0.011	0	20	*ple*
0	4.266282	0.927	0.022	0	20	*sNPF*
0	3.807545	0.996	0.226	0	20	*klg*
0	3.187185	0.998	0.448	0	20	*DAT*
0	3.084102	0.853	0.097	0	20	*Ets65A*
0	2.553052	0.722	0.104	0	20	*CG45263*
0	2.28098	0.897	0.454	0	20	*beat-IIIb*
0	2.277949	0.947	0.44	0	20	*CG8861*
0	2.249176	0.92	0.374	0	20	*CG42342*

C

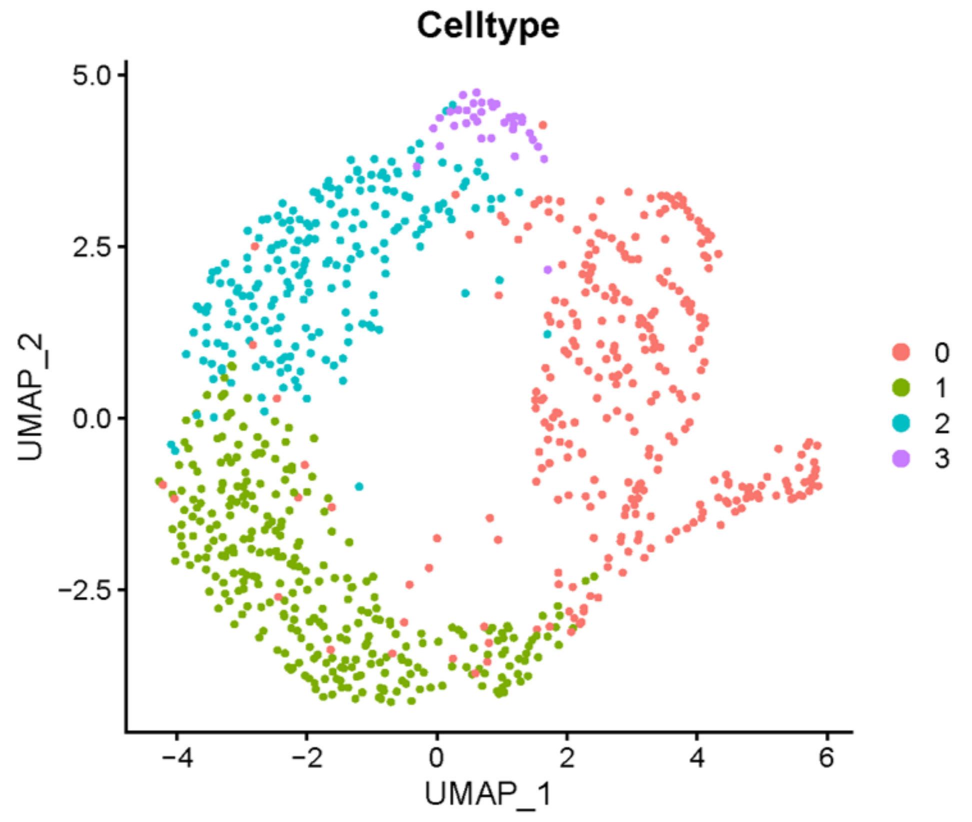

Supplement: Supplementary file 12 — Supplementary Figure 3 [file 41380_2023_2314_MOESM12_ESM.pdf]
